# RNA‐centric world of retroviruses: unravelling the molecular strategies of genomic RNA packaging

**DOI:** 10.1002/brv.70188

**Published:** 2026-05-26

**Authors:** Mohammad Abdullah Jehad, Suresha G. Prabhu, Lizna M. Ali, Vineeta N. Pillai, Farah Mustafa, Tahir A. Rizvi

**Affiliations:** ^1^ Department of Microbiology & Immunology, College of Medicine and Health Sciences (CMHS) United Arab Emirates University (UAEU) Al Ain 15551 UAE; ^2^ Department of Biochemistry & Molecular Biology, College of Medicine and Health Sciences (CMHS) United Arab Emirates University (UAEU) Al Ain 15551 UAE; ^3^ Zayed bin Sultan Center for Health Sciences (ZCHS) United Arab Emirates University Al Ain Al Ain UAE

**Keywords:** Retroviruses, Retroviral Replication, RNA Structure & Function, RNA dimerization, RNA packaging, RNA‐protein Interaction, unpaired or Single Stranded Purines, long‐range RNA–RNA Interactions (LRIs), targets of anti‐viral therapies

## Abstract

Retroviruses constitute a unique group of RNA viruses that have profoundly influenced both evolutionary trajectories and biomedical research. Their ability to reverse transcribe and integrate into host genomes has shaped genomic architecture across species and contributed to our understanding of oncogenes, gene regulation, and RNA biology. This review provides an in‐depth examination of retroviral molecular biology, beginning with the intricacies of their genome organization. We present an in‐depth discussion of the complex interplay of *cis*‐ and *trans*‐acting RNA elements during retrovirus replication, ending with a focus on genomic RNA packaging as the basis of infectious virion production. Emphasis is placed on how, among the various viral proteins, it is the retroviral Gag (Group‐specific antigen) that selectively orchestrates the packaging of the unspliced RNA amid a background of cellular and spliced viral RNAs. This is followed by our current understanding of how this process is affected by the retroviral Gag/Pol (Polymerase) and Gag/Pro (Protease)/Pol precursor proteins in the context to which the actual virion assembly takes place. Next, the review outlines emerging concepts regarding the structural basis of RNA recognition, the pivotal role of unpaired purines and long‐range intragenomic interactions (LRIs), in guiding genome selection. It discusses the dynamic processes of RNA dimerization and how it is not just intricately linked but is a pre‐requisite for genomic RNA encapsidation. Special attention is given to the new findings on transcription start site heterogeneity, cap sequestration, and epitranscriptomic modifications in shaping the fate of retroviral RNAs between translation and packaging since both these processes are critical for a successful viral life cycle. The review also addresses the involvement of assembly intermediates, and liquid–liquid phase separation in orchestrating efficient genome packaging and virus maturation. By integrating classical discoveries with recent findings, the review underscores the sophistication of retrovirus replication and proposes new avenues of research for therapeutic targeting of genome packaging and steps of viral assembly.

## I. INTRODUCTION

Retroviruses are a fascinating group of viruses that have significantly influenced the biological and evolutionary landscape of virology. Retroviruses exhibit the ability to infect various species across the animal kingdom, with a notable presence in mammals, including humans (Goff, 2013; Skalka, 2018). They are associated with several diseases, such as immunodeficiency syndromes and a variety of cancers, both hematological and solid tumours. Throughout the twentieth century, retroviruses played a pivotal role in biological discoveries, unveiling phenomena such as cell transformation, viral and cellular oncogenes, reverse transcription, and viral transduction. These breakthroughs paved the way for complementary DNA (cDNA) cloning and the development of retroviral vectors for human gene therapy (Telesnitsky & Goff, 1997). In contrast to the positive‐sense RNA viruses found in many animals, retroviral genomes are thought not to serve directly as mRNAs, and consequently, they are not directly translated to make viral proteins. Rather than relying on direct translation, retroviruses utilize the virally encoded reverse transcriptase (RT) to reverse transcribe their genomic RNA (gRNA) into a full‐length viral DNA intermediate. This DNA intermediate, called cDNA, is subsequently integrated into the cellular host DNA mediated by the virally encoded integrase (IN) enzyme, creating proviral DNA (Telesnitsky & Goff, 1997). By integrating their genetic material into the host genome, retroviruses not only propagate their lineage but also leave a lasting imprint on the host's genetic makeup. This is despite the fact that retroviral gRNA, like other host cellular mRNAs and other positive‐sense viral RNAs, are methylated, capped, and contain polyadenylation sequences, hallmarks of translationally competent mRNAs (Telesnitsky & Goff, 1997). While a recent report claims that the retroviral genome can be directly translated (Köppke *et al*., 2024), the widely accepted belief still holds that retroviruses primarily undergo reverse transcription and integration prior to translation of their mRNAs from the provirus.

The biggest contribution of retroviruses to the field of medicine has been in the development of vectors for human gene therapy and vaccines, areas that have revolutionized molecular medicine (Chen, Saubi & Joseph‐Munné, 2020; Morgan *et al*., 2021). The development of these fields required a better understanding of how retroviruses replicate and package their genome into the assembling virus particle. Given the fact that the retroviral genome or gRNA is virtually ~1% or less when compared to the total mRNA present in the infected cell, retroviruses must face challenges during selection for packaging/encapsidation amid a high background of host cellular and viral sub‐genomic RNAs (Ali, Rizvi & Mustafa, 2016; Bieniasz & Telesnitsky, 2018; Chameettachal, Mustafa & Rizvi, 2023; Comas‐Garcia, Davis & Rein, 2016; D'Souza & Summers, 2005; Dubois *et al*., 2018; Johnson & Telesnitsky, 2010; Kutluay *et al*., 2014; Lever, 2007; Lu *et al*., 2011, 2011; Maldonado & Parent, 2016; Miyazaki *et al*., 2011; Rulli *et al*., 2007). The ability of retroviral structural proteins to specifically choose retroviral genome from a surplus of heterologous RNAs suggests the presence of specific sequences within the gRNA which play a role in directing the specific and efficient packaging of retroviral gRNAs into the virus particles. Over the last five decades, hundreds of studies have revealed the existence of *cis*‐acting sequences, commonly referred to as *psi* (ψ), involved in this specific RNA packaging process. These sequences are typically found at the 5′ end of the retroviral genome (Ali *et al*., 2016; Berkhout & van Wamel, 1996; Bieniasz & Telesnitsky, 2018; Browning *et al*., 2003; Chameettachal *et al*., 2023; Clever, Miranda & Parslow, 2002; Comas‐Garcia *et al*., 2016; Das *et al*., 1997; D'Souza & Summers, 2005; Dunbar *et al*., 2018; Johnson & Telesnitsky, 2010; Kenyon *et al*., 2008; Kutluay *et al*., 2014; Lever, 2007; Lu *et al*., 2011, 2011; Mailler *et al*., 2016; Maldonado & Parent, 2016; Miyazaki *et al*., 2011; Mustafa *et al*., 2005; Rulli *et al*., 2007; Rizvi *et al*., 2010; Smyth *et al*., 2018).

Detailed identification and functional demarcation of retroviral RNA packaging signals have enabled the rational engineering of viral assembly systems for biotechnology applications. By separating the *cis*‐acting packaging elements (such as Ψ and associated purine‐rich, single‐stranded motifs) from coding sequences, investigators have generated retroviral‐based vectors in which therapeutic or reporter RNAs are selectively packaged, while viral structural proteins are supplied in *trans* by packaging cell lines. This modularization underlies the safety and efficiency of modern gene therapy vectors, as it minimizes the risk of replication‐competent virus formation while preserving high‐level vector RNA packaging and particle production (Naldini *et al*., 1996; Naldini, 2011, 2015; Milone & O'Doherty, 2018; Buckingham *et al*., 2024; Jaballah *et al*., 2025). Furthermore, targeted disruption or omission of packaging sequences has been exploited to produce virus‐like particles (VLPs) that assemble and bud efficiently, yet are devoid of gRNA, allowing their use as non‐infectious immunogens (Chen *et al*., 2020; Morgan *et al*., 2021). Such gRNA‐deficient VLPs have proven valuable for vaccine development, where they present native viral antigens in a highly immunogenic context. Similarly, such VLPs have also been used as delivery platforms for heterologous RNAs for nucleic acid delivery strategies, where controlled RNA packaging can be tuned to optimize stability, expression, and safety. Collectively, these advances highlight how mechanistic understanding of retroviral genome packaging has been directly translated into versatile platforms for human gene therapy and vaccine design. However, there are still gaps in our understanding about the exact secondary and/or tertiary structure and specificity of these *cis*‐acting packaging sequences. Moreover, the understanding of viral gene product(s) that specifically interact with the packaging sequences to ensure proper packaging of the retroviral genome for infectious virus particle production remains limited. While it is clear that *gag* gene products are necessary for gRNA packaging, the intricacies of the RNA–protein interaction(s) involved in this process remain largely unknown (Ali *et al*., 2016; Chameettachal *et al*., 2023; Comas‐Garcia *et al*., 2016; D'Souza & Summers, 2005; Dubois *et al*., 2018; Johnson & Telesnitsky, 2010; Lever, 2007; Maldonado & Parent, 2016).

Despite major advances in identifying retroviral proteins, *cis*‐acting RNA elements, and the host factors exploited by retroviruses, understanding the molecular underpinnings that govern retrovirus replication, and in particular retroviral gRNA packaging, remains a challenge. Remarkably, retroviruses have evolved complex structural strategies to selectively package their genomic RNA in a cellular environment packed with other viral and host RNAs. This review highlights the current knowledge of the molecular intricacies of retroviral genome, followed by an in‐depth review of genomic RNA packaging, focusing on the role of RNA structural elements, dynamics of RNA dimerization, and host‐mediated processes, including transcription site heterogeneity and epitranscriptomic modifications. By integrating classical discoveries with emerging insights, it highlights established mechanisms and unresolved questions that continue to drive the field. A deeper understanding of retroviruses and genomic RNA packaging holds promise for not only advancing antiviral therapeutic strategies but also improving the design of retroviral vectors for gene therapy.

## II. RETROVIRAL GENOME ORGANIZATION

As illustrated in Fig. 1, the RNA genome of retroviruses can be broadly classified into *cis*‐ and *trans*‐acting sequences/elements. The *cis*‐acting elements comprise of non‐coding regulatory sequences, whereas the *trans*‐acting elements are sequences coding for the structural and functional/enzymatic proteins of the virus (Fig. 1; Coffin, Hughes & Varmus, 1997; Knipe & Howley, 2013). *The cis*‐acting elements are generally confined to the 5′ and 3′ ends of the viral RNA genome, while the *trans*‐acting elements occupy the center of the retroviral/proviral genome.

**Fig. 1:**
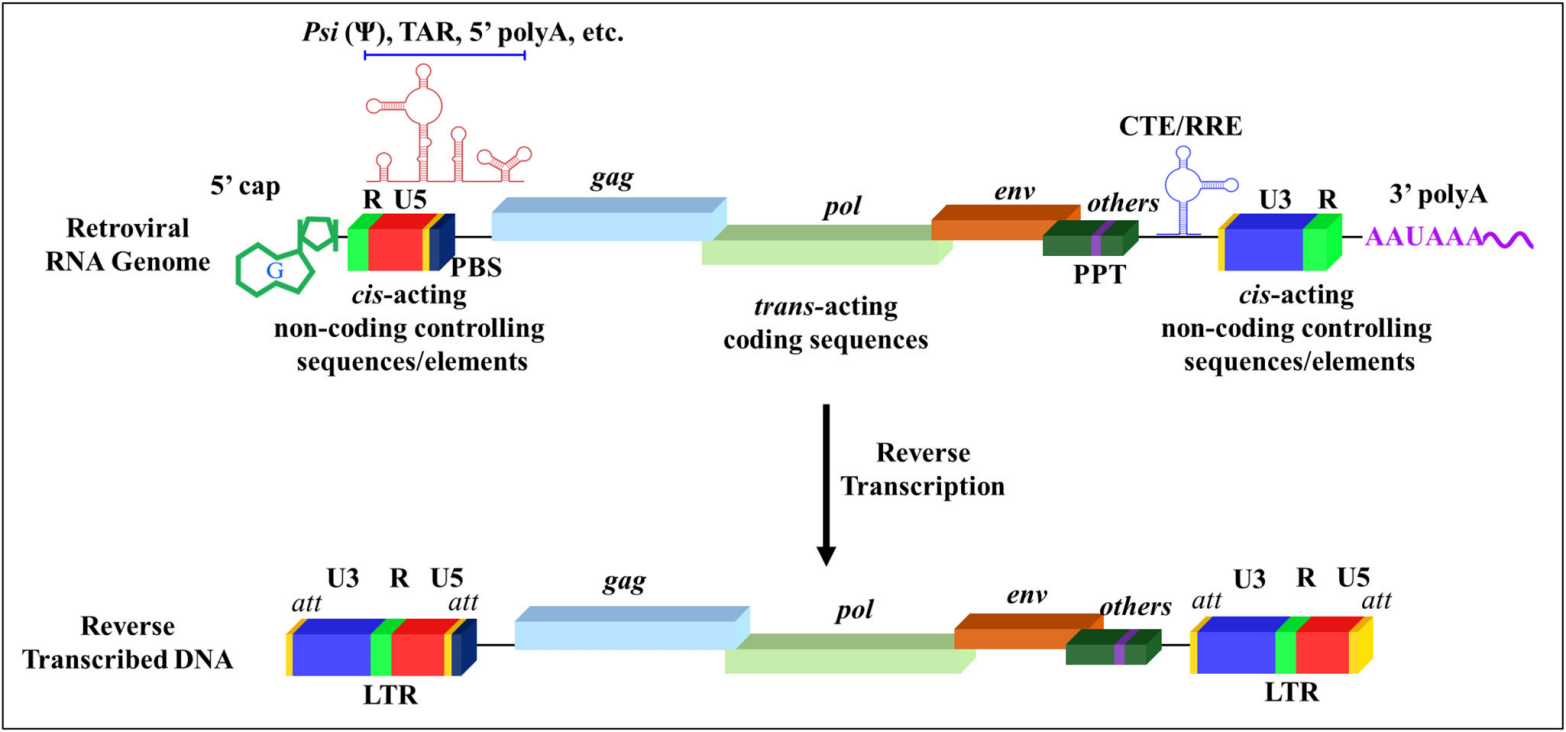
Schematic representation of the retroviral RNA genome and its reverse‐transcribed DNA highlighting the arrangement of *cis*‐ (non‐coding) and *trans*‐acting (coding) sequences. *att*, attachment sites of the virally encoded integrase enzyme; CTE, the constitutive transport element specific to Mason‐Pfizer monkey virus (MPMV); PBS, primer binding site; PPT, polypurine tract; *Psi* (Ψ), packaging sequences or signals; R, repeat regions; RRE, the Rev response element specific to HIV‐1; TAR, the *trans*‐activation response element specific to human immunodeficiency virus type 1 (HIV‐1); U5 & U3, sequences unique to the 5′ and 3′ ends of the RNA genome.

### (1) *Cis*‐acting non‐coding sequences and/or elements

The repeat region generally known as R is found at both the 5′ and 3′ ends of the viral genome and plays a key role during reverse transcription and, in some viruses, transcriptional regulation (e.g. the *trans*‐acting region, TAR, in *lentiviruses*). Transcription is initiated at the start of the 5′ R region (often referred to as +1R) and continues until the end of the 3′ R region. The R region has a fundamental role in reverse transcription. The ‘unique’ sequence/region located immediately after the R but before the beginning of the primer binding site (PBS) at the 5′ end is termed as ‘U5’ (a region unique to the 5′ end; Fig. 1). Similarly, the region located immediately upstream of R at the 3′ end is termed ‘U3’ (a region unique to the 3′ end) (Coffin *et al*., 1997; Hunter, 2008; Knipe & Howley, 2013). These U5 and U3 regions also contain the attachment (*att*) sites required for the integration of the reverse‐transcribed RNA into the host genome (see the U5 and U3 *att* sites in Fig. 1; Coffin *et al*., 1997; Hunter, 2008; Knipe & Howley, 2013; Kulkosky & Skalka, 1994; Masuda, Kuroda & Harada, 1998; Roth, Schwartzberg & Goff, 1989; Sherman & Fyfe, 1990). During reverse transcription of retroviral RNA, the U5 and U3 regions are duplicated and relocated to the 3′ and 5′ ends of the genome, forming the complete long terminal repeats (LTRs; Fig. 1). Finally, the 3′ end of the viral genome, overlapping the 3′ U3 region, also contains *cis*‐acting structural RNA elements important for nucleocytoplasmic transport of retroviral unspliced genomic RNA [e.g. the Rev responsive element (RRE) in *lentiviruses* or constitutive transport element (CTE) in simple retroviruses; Fig. 1].

The PBS is ~18 nucleotides (nts) long and is highly conserved. It is present downstream of U5 and binds specific transfer RNAs (tRNAs) to initiate reverse transcription (Coffin *et al*., 1997; Hunter, 2008; Isel, Ehresmann & Marquet, 2010; Knipe & Howley, 2013; Marquet *et al*., 1995). The polypurine tract (PPT) is present upstream of U3 and resists RNase H cleavage. It serves as a primer for plus‐strand DNA synthesis during reverse transcription (Coffin *et al*., 1997; Huber & Richardson, 1990; Rausch & Le Grice, 2004).

### (2) *Trans*‐acting coding sequences and/or regions

The central part of the retroviral genome flanked by the *cis*‐acting sequences is often referred to as the *trans*‐acting sequences/regions and contains the coding regions for viral structural (group‐specific antigen, Gag) and enzymatic [protease (Pro) or PR & polymerase (Pol)], and envelope (Env) proteins (Fig. 1). The Gag polyprotein consists of three major domains found in all retroviruses, in the order from the 5′ end: matrix (MA), capsid (CA), and nucleocapsid (NC), essential for core formation and virion particle production. In addition, it could have several other spacer peptides and other domains that differ among retroviruses with functional consequences for virion particle production. The Pro/PR encodes for the viral protease enzyme, required for the proteolytic cleavage of the Gag, Pro, and Pol precursor proteins. Finally, Pol is the biggest gene of the virus encoding for the RT, IN, and RNase H, enzymes crucial for the important processes of reverse transcription and integration.

In addition to these highly conserved and essential genes, retroviral genomes can encode multiple other factors that play regulatory or accessory roles in the viral life cycle, such as Tat and Rev or their equivalent, essential for virus transcriptional regulation and nuclear export observed in *beta*, *delta* and *lentiviruses*, or unique like the Superantigen (Sag), dispensable for viral life cycle, but essential for virus spread in the host and immune response to the viral infection in mouse mammary tumour virus (MMTV) (Coffin *et al*., 1997).

## III. A SNAPSHOT OF THE RETROVIRAL LIFE CYCLE

The life cycle of retroviruses comprises of a complex series of events that allow them to replicate and spread within their host. Each step of the viral life cycle is orchestrated by specific viral proteins and enzymes, enabling the virus to hijack the host cell machinery, specifically its proteins/enzymes, for its own replication. The retroviral life cycle can be divided into two phases: The first phase involves entry, reverse transcription, and integration into the host genome, all of which utilize virally encoded proteins and enzymes. The second phase includes transcription (synthesis of viral genomes and mRNAs) and translation (synthesis of viral proteins), which rely on the host cell's machinery. Major steps during the retroviral life cycle are briefly discussed below and outlined in Fig. 2.

**Fig. 2:**
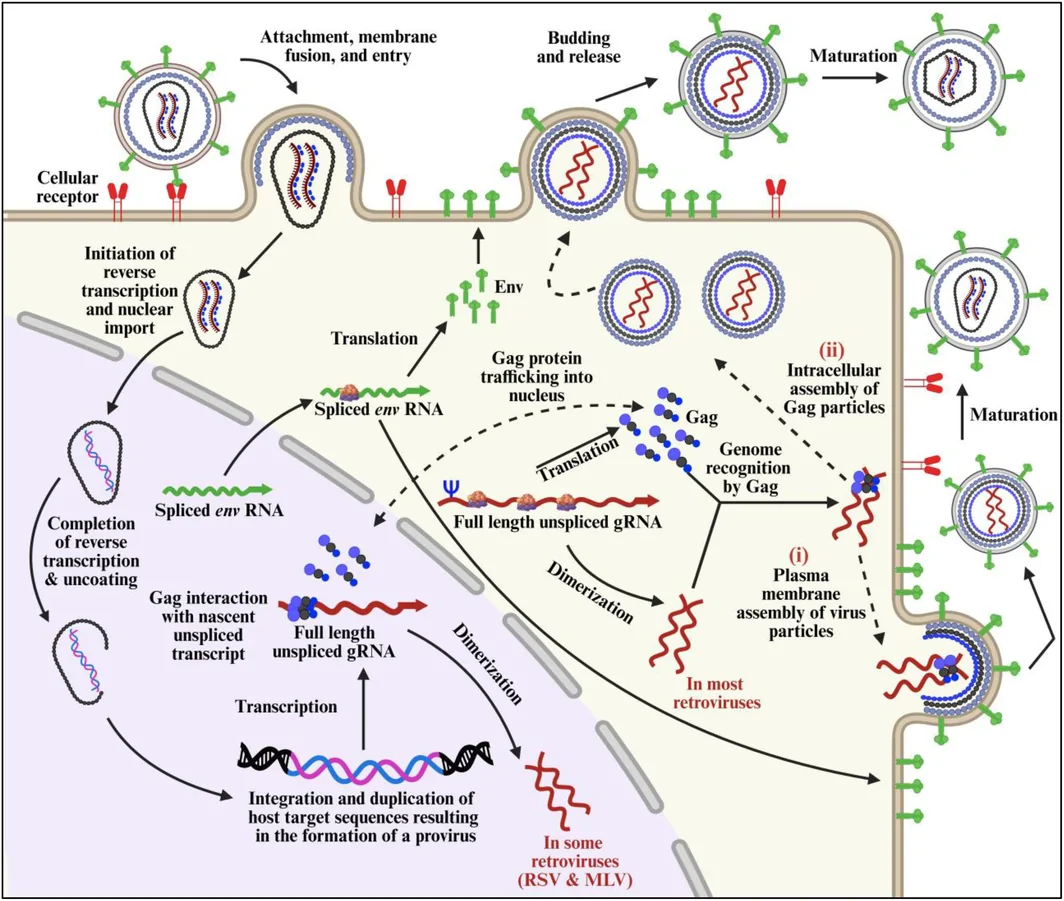
Schematic depiction of the various stages of retroviral replication, highlighting both plasma membrane and intracellular assembly modes. Following receptor‐mediated attachment and entry into a susceptible host cell, the viral core is released into the cytoplasm. In all retroviruses, reverse transcription of the viral RNA is initiated within the core in the cytoplasm. Recent studies indicate that, at least in human immunodeficiency virus type 1 (HIV‐1) and murine leukemia virus (MLV), the core enters the nucleus where reverse transcription is completed, uncoating occurs, and the reverse‐transcribed DNA is integrated into the host genome to form a provirus. Transcription of the provirus is carried out by the host cell's machinery. The resulting full‐length unspliced and spliced RNAs are exported from the nucleus. The full‐length unspliced RNA serves dual functions: it acts as the template for translating Gag, Pro, and Pol proteins while also functioning as the genomic RNA (gRNA) that dimerizes and binds to the Gag polyprotein for packaging into newly assembling virions. Virion assembly can occur (*i*) either directly at the plasma membrane (type‐C), or (*ii*) through pre‐assembly in the cytoplasm (type‐B/D) followed by transport to the plasma membrane where budding virus particles acquire the envelope glycoproteins. Finally, immature viral particles are released from the cell, and maturation is completed post‐release through proteolytic cleavage and structural rearrangement of viral components. The figure also illustrates the nuclear population of Gag, the interaction of Gag with unspliced nascent viral gRNA, as well as gRNA dimerization in the nucleus, as has been documented for some retroviruses. MLV, murine leukemia virus; RSV, Rous sarcoma virus.

As mentioned earlier, retroviruses display different modes of virion assembly that can be either at the plasma membrane level (e.g. type‐C) or intracellularly within the cytoplasm, as seen in type‐B/D retroviruses. The reason why some retroviruses follow the type‐C assembly mode while others adopt the type‐B/D assembly mode (Fig. 2) remains largely unclear. However, it is well‐known that the key factors driving retroviral assembly are encoded within their Gag polyprotein. Even in the absence of other viral components, especially protease, expressing MMTV, feline immunodeficiency virus (FIV), and simian immunodeficiency virus (SIV) Gag polyproteins independently directs the virus to the appropriate assembly pathway (Krishnan *et al*., 2019; Pillai *et al*., 2023; Zábranský *et al*., 2009). The critical role of Gag in virion assembly has been clearly demonstrated by amino acid substitutions(s) in the MA sequence of Mason‐Pfizer monkey virus (MPMV), MMTV, Jaagsiekte sheep retrovirus (JSRV), and *spumaviruses*, resulting in the relocation of their assembly from cytoplasm to the plasma membrane – a major shift from a type D to a type‐C pattern (Murcia, Arnaud & Palmarini, 2007; Rhee & Hunter, 1990; Zhang *et al*., 2015). The MA domain of these retroviruses includes a ‘cytoplasmic targeting and retention signal’ (CTRS), which is essential for assembling virus particles within the pericentriolar region of the cytoplasm. Amino acid change in the CTRS can shift capsid assembly from the cytoplasm to the plasma membrane, highlighting the critical role of Gag in both virus‐like particle (VLP) formation and the precise intracellular positioning of retrovirus assembly (Choi *et al*., 1999; Sfakianos, LaCasse & Hunter, 2003; Yu, Eastman & Linial, 2006). Along the same lines, alterations in the MA domain of human immunodeficiency virus type 1 (HIV‐1) Gag, whether through amino acid substitutions or deletions, have been shown to redirect virus assembly to the cytoplasm or intracellular membranes, such as the endoplasmic reticulum (Cannon *et al*., 1997; Fäcke *et al*., 1993). These observations suggest that, despite differences in assembly modes, the functions governed by Gag subdomains across various retroviruses may be more similar than previously assumed (Junková *et al*., 2020).

### (1) Genomic RNA (gRNA) packaging

Retroviral gRNA packaging and virus assembly are intricately linked processes that are crucial for generating infectious virions. During assembly, the Gag polyprotein plays a central role in selectively packaging/encapsidating gRNA. This specificity is mediated by interactions between the Gag polyprotein and a *cis*‐acting packaging sequences (*Psi*; ψ). The retroviral *Psi* sequences have been mapped to regions within the 5′ untranslated region (UTR), often extending into the *gag* gene, a region located within the major intron of the viral RNA; that is the unspliced RNA (Ali *et al*., 2016; Berkhout & van Wamel, 1996; Bieniasz & Telesnitsky, 2018; Chameettachal *et al*., 2023; Clever *et al*., 2002; Comas‐Garcia *et al*., 2016; Das *et al*., 1997; D'Souza & Summers, 2005; Dubois *et al*., 2018; Jaballah *et al*., 2010; Johnson & Telesnitsky, 2010; Jouvenet *et al*., 2011; Kenyon *et al*., 2008; Maldonado & Parent, 2016; Miyazaki *et al*., 2011; Mustafa *et al*., 2005, 2012; Rulli *et al*., 2007; Rizvi *et al*., 2010; Smyth *et al*., 2018). This ensures that the unspliced gRNA is preferentially incorporated over the vast majority of cellular and spliced viral RNAs, as depicted in a very simplistic model in Fig. 3. The two copies of gRNA interact with each other to form a dimeric RNA, a step that is proposed to be a prerequisite for encapsidation into the assembling virions (Moore & Hu, 2009; Paillart *et al*., 2004a). While a single gRNA can serve as a template for reverse transcription and proviral DNA synthesis, the characteristic recombination and genetic reassortment of retroviruses rely on the presence of a diploid genome (Hu & Temin, 1990). Therefore, prior to packaging, two full‐length gRNAs anneal, thus initiating the formation of loose, noncovalent RNA dimers, which are then stabilized within assembling virions. Simultaneously, Gag polyprotein oligomerization drives virus particle formation, budding, and maturation. Such precise coordination between RNA selection and structural assembly ensures the production of replication‐competent virions.

**Fig. 3:**
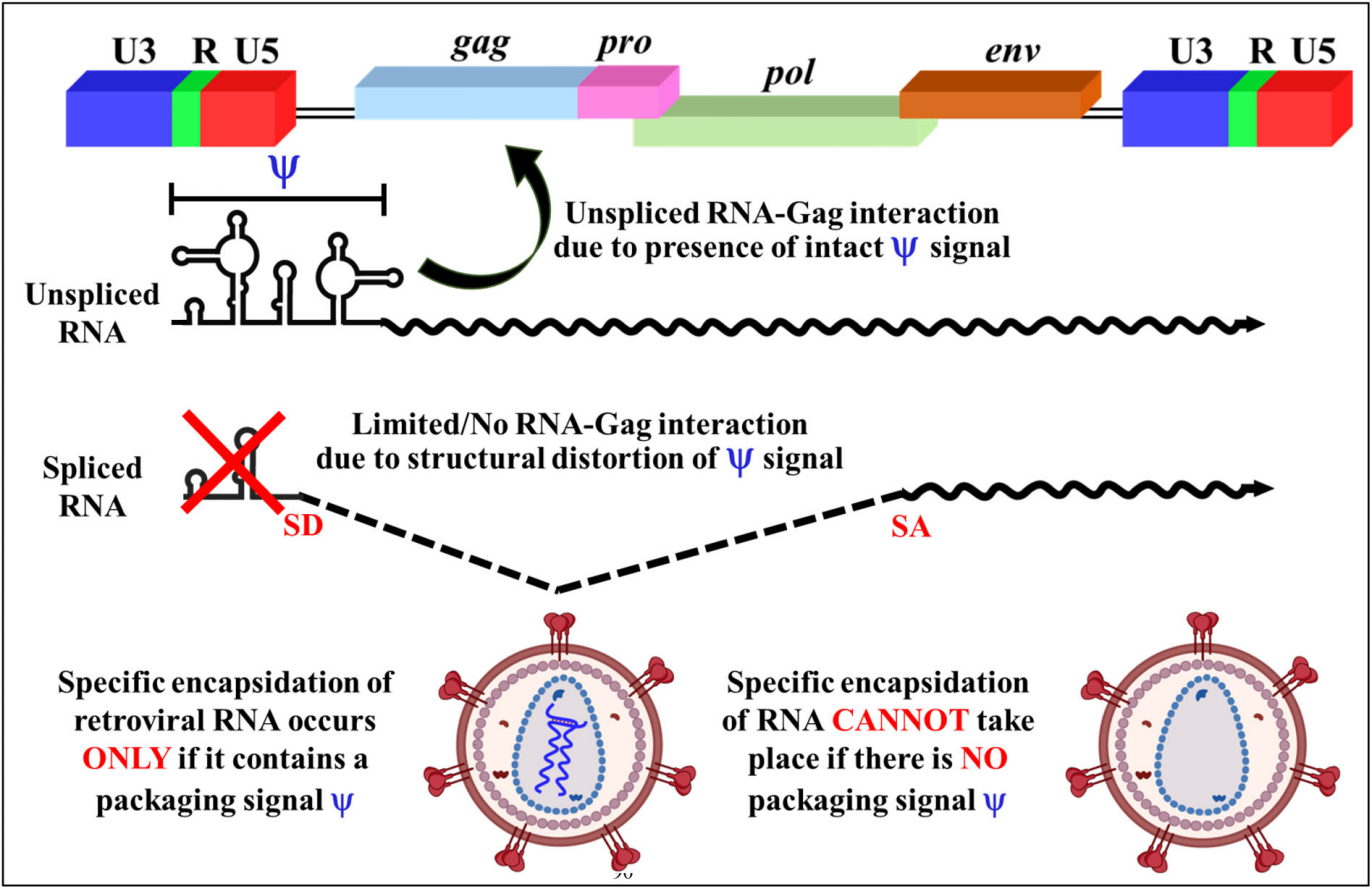
Graphical representation of a simplistic retroviral genomic RNA (gRNA) packaging model illustrating selective recognition of gRNA by Gag based on recognition of the higher‐order structure of the *cis*‐acting packaging sequences (*Psi*; ψ). These structural motifs expose specific nucleotides serving as key contact points for Gag binding facilitating packaging of the unspliced RNA. During splicing, the Gag binding nucleotides in the packaging sequences are either removed or their structural conformation is disrupted, preventing efficient Gag–RNA interactions and limiting the packaging of the spliced RNA into the assembling virions. R, repeat regions; SA, splice acceptor; SD, splice donor; U5 & U3, sequences unique to the 5′ and 3′ ends of the RNA genome.

In contrast to most retroviruses which contain packaging signals primarily on the unspliced RNA, in Rous sarcoma virus (RSV) (and HIV‐2), the packaging signal is present on both the spliced and unspliced viral RNAs (Katz *et al*., 1986; Banks, Kealoha & Linial, 1999; Griffin, Allen & Lever, 2001), raising the obvious question of what dictates specificity of packaging of the genomic, unspliced RNA. In RSV, this specificity is thought to arise from a combination of mechanisms rather than a single determinant (Maldonado & Parent, 2016). Unspliced RNA is translated on free cytoplasmic ribosomes, and may be co‐translationally captured by newly synthesized Gag, whereas spliced Env mRNAs are sequestered into the endoplasmic reticulum where they are inaccessible for packaging (Donzé & Spahr, 1992; Donzé, Damay & Spahr, 1995). Temporal regulation may further bias RNA fate, with early transcripts favouring translation until sufficient Gag accumulates to initiate encapsidation (Sonstegard & Hackett, 1996). In addition, the RSV packaging sequences in the unspliced RNA adopt a distinct higher‐order leader structure (Banks *et al*., 1998) that could potentially expose structural motifs, such as long‐range RNA–RNA interactions. This in turn could allow the unspliced RNA to dimerize more efficiently, promoting high‐affinity Gag binding and packaging (Maldonado & Parent, 2016). Finally, differential association of host splicing factors with spliced RNAs may further inhibit their encapsidation (Knoener *et al*., 2021). Together, these differences in the context of translation, timing, RNA structure, dimerization, and host factor binding offer a coherent framework for how RSV may selectively package gRNA despite shared packaging signals on both unspliced and spliced RNAs. The same is true for HIV‐2, where several studies point towards similar potential mechanisms for selectively packaging unspliced over spliced RNA for encapsidation into the viral genome (Kaye & Lever, 1999; Griffin *et al*., 2001; Lanchy *et al*., 2003a; Balvay *et al*., 2007; Baig, Lanchy & Lodmell, 2007).

### (2) Recognition of packaging sequences by gag is on a structural basis rather than at the sequence level

A long‐standing question in retrovirology has been what makes the packaging sequences of retroviruses uniquely capable of preferentially binding to Gag over other RNA sequences. The specificity of retroviral RNA packaging is not solely determined by primary sequence recognition, but also by secondary and tertiary RNA structures. This was evident from studies showing that packaging specificity can be exchanged between phylogenetically diverse retroviruses that lack sequence homology. For example, the 5′ region of murine leukemia virus (MLV) and certain other retroviruses have been identified as both necessary and sufficient for gRNA packaging. When these sequences are incorporated into heterologous or even non‐retroviral RNAs, they enable the efficient packaging of these foreign RNAs into assembling virions (Adam & Miller, 1988; Al Dhaheri *et al*., 2009; Al Shamsi *et al*., 2011; Hibbert, Mirro & Rein, 2004). This structural‐based recognition mechanism explains the phenomena of co‐packaging and cross‐packaging observed between phylogenetically divergent retroviruses those have no sequence homology (Al Dhaheri *et al*., 2009; Al Shamsi *et al*., 2011; Browning *et al*., 2001; Certo *et al*., 1998; Das *et al*., 1997; Embretson & Temin, 1987; Rizvi & Panganiban, 1993; White *et al*., 1999; Yang & Temin, 1994; Yin & Hu, 1997). Further supporting this model, specific nucleotides within these structured motifs have been shown to bind Gag precursor polyproteins with high specificity in various retroviruses, including HIV‐1, MPMV, MMTV, and FIV (Abd El‐Wahab *et al*., 2014; Bernacchi *et al*., 2017; Chameettachal *et al*., 2021; Didierlaurent *et al*., 2011; Krishnan *et al*., 2023; Pitchai *et al*., 2021; Prabhu *et al*., 2024; Smyth *et al*., 2015, 2018). These interactions occur within the structural context of the RNA, reinforcing the idea that packaging is driven by higher‐order RNA folding rather than simple sequence recognition.

Over the years, it has now been well‐established that retroviral packaging sequences indeed fold into higher‐order structures (Aktar *et al*., 2013, 2014; Chameettachal *et al*., 2023; Clever, Sassetti & Parslow, 1995; Comas‐Garcia *et al*., 2016; D'Souza & Summers, 2004, 2005; Dubois *et al*., 2018; Jaballah *et al*., 2010; Johnson & Telesnitsky, 2010; Kenyon *et al*., 2008, 2011; Lever, 2007; Maldonado & Parent, 2016; Mustafa *et al*., 2018; Rizvi *et al*., 2010; Zeffman *et al*., 2000). These structural motifs expose specific nucleotides that serve as key contact points for Gag binding, thereby facilitating the selective packaging of retroviral gRNA. The simplified model, as depicted in Fig. 3, can partially explain how full‐length unspliced gRNA is preferentially packaged over spliced viral RNAs. During splicing, the packaging sequences may be removed, or their structural conformation may be disrupted, as has been reported for MMTV, MPMV, and FIV (Chameettachal *et al*., 2021; Krishnan *et al*., 2023; Pitchai *et al*., 2021), preventing efficient Gag–RNA interactions and subsequent packaging into the assembling virions. The possibility of removal of the core packaging element in the case of HIV‐1 has been proposed based on nuclear magnetic resonance (NMR) studies (Ding *et al*., 2020). Ding *et al*. (2020) have identified a (UUUU):(GGAG) helical stem in a three‐way junction structure as an initial high‐affinity binding site for the Gag NC domain. However, since the ‘GGAG’ is present downstream of the major splice donor, mSD, it can be spliced out (Ding *et al*., 2020). Conversely, in full‐length unspliced gRNA, the intact higher‐order structure remains preserved, enabling optimal Gag binding and selective packaging. This structural‐based discrimination provides a plausible mechanism by which retroviruses distinguish between spliced and unspliced viral RNAs during the packaging process.

### (3) Role of NC and full‐length gag in recognition of packaging sequences

The NC domain of Gag plays a key role in selective retroviral gRNA packaging (Ali *et al*., 2016; Dubois *et al*., 2018; Lever, 2007; Lu *et al*., 2011b; Maldonado & Parent, 2016; Rawson *et al*., 2022). This is primarily because of its basic nature harbouring Cys‐His boxes that can interact with Zn^2+^ ions to facilitate protein/RNA interactions. Consistent with this, mutations in the zinc fingers significantly impair gRNA packaging and result in the formation of non‐infectious virions (Aldovini & Young, 1990; Gorelick *et al*., 1988; Wang & Barklis, 1993). However, the full‐length Gag exhibits greater specificity in binding to the packaging sequences compared to the NC domain, indicating that additional Gag domains contribute to the retroviral gRNA packaging process (Gherghe *et al*., 2010). Thus, besides NC, other domains of the Gag protein contribute to the recognition of the packaging sequences. For instance, in HIV‐1, these include MA (Ott, Coren & Gagliardi, 2005; Ristic & Chin, 2010), CA (Carlson *et al*., 2016; Guo *et al*., 2005; Lei *et al*., 2023), p6 (Dubois *et al*., 2018), and the spacer peptide sp1 (Ristic & Chin, 2010; Russell *et al*., 2003), all of which assist in ensuring specific and efficient gRNA encapsidation by Gag. This is further substantiated by the fact that substituting the RSV NC domain with MLV or replacing the HIV‐1 NC domain with NC of MMTV did not entirely abolish RNA packaging; instead, up to 50% of RNA packaging was preserved (Dupraz & Spahr, 1992; Poon, Li & Aldovini, 1998).

Despite extensive studies on retroviral RNA packaging, a comprehensive understanding of how retroviral genomes are selectively packaged during assembly remains elusive. This is primarily due to the unavailability of a functionally active full‐length Gag protein responsible for the initial interaction with packaging sequences on unspliced gRNA. Recent studies in HIV‐1 prove the pivotal role of the full‐length Gag precursor in selective recognition and packaging of gRNA from a pool of host cellular and spliced viral RNAs (Abd El‐Wahab *et al*., 2014; Bernacchi *et al*., 2017; Didierlaurent *et al*., 2011; Smyth *et al*., 2015, 2018). Earlier studies on HIV‐1 RNA packaging focused solely on the NC domain of Gag and identified a GGAG tetraloop within stem‐loop 3 (SL3) of viral RNA as a high‐affinity Gag binding site. This led to a misleading conclusion that SL3 served as the primary and sole packaging determinant of HIV‐1 (De Guzman *et al*., 1998; Ding *et al*., 2020; Keane *et al*., 2015; Kenyon, Prestwood & Lever, 2015). However, studies using the full‐length HIV‐1 Gag demonstrated that gRNA packaging is instead facilitated by a G//AGG internal loop within SL1, rather than the GGAG tetraloop in SL3 (Abd El‐Wahab *et al*., 2014; Bernacchi *et al*., 2017; Smyth *et al*., 2015, 2018). Despite these advances in HIV‐1 research, similar RNA‐binding investigations were missing for other retroviruses due to a lack of purified full‐length Gag precursor proteins. Recent RNA–protein interaction studies utilizing full‐length Gag from MMTV, MPMV, and FIV have successfully identified specific nucleotides that exhibit high‐affinity binding to their respective Gag proteins in the context of their secondary RNA structures (Chameettachal *et al*., 2021; Krishnan *et al*., 2023; Pitchai *et al*., 2021; Prabhu *et al*., 2024). These findings highlight the critical need to study retroviral gRNA packaging using the full‐length Gag precursor polyprotein to fully elucidate the mechanisms underlying selective gRNA packaging.

### (4) Role of gag‐pol, gag‐pro, and gag‐pro‐pol precursors in recognition of packaging sequences

Considering the above‐mentioned studies, it is both reasonable and important to question whether full‐length Gag alone is sufficient for RNA packaging, or whether the Pro and Pol domains also play a role, especially since RNA packaging occurs in the context of the Gag‐Pol or Gag‐Pro‐Pol precursors, rather than Gag alone. Immature retroviral particles incorporate ~2,000 copies of the Gag precursor, and ~100 copies of Gag‐Pol precursors (in HIV‐1 and FIV) or Gag‐Pro and Gag‐Pro‐Pol precursors (in MMTV and MPMV), which generate viral enzymes after maturation of the viral particles, in addition to Gag. One of the earliest studies to suggest a link between Gag/Pol precursors and gRNA packaging came from the study of Dougherty and colleagues (Kobayashi *et al*., 2010) who showed that knockdown of the translation termination factor, eRF1, led to the formation of non‐infectious HIV‐1 viral particles that they attributed to an increase in the ratio of Gag‐Pol to Gag mRNA in infected cells. Later on, this led to the discovery of the Gag RNA Packaging Enhancer (GRPE) observed within the p6 domain of HIV‐1 Gag which reduces gRNA packaging by 50% when deleted or mutated (Chamanian *et al*., 2013). Interestingly, GRPE overlaps with the ribosomal frameshift site of Gag‐Pol, suggesting that translation termination may be linked to gRNA packaging, an observation that was later confirmed by knockdown of eRF1 which led to the formation of defective viral particles with >20 times more gRNA than normal. This led to the hypothesis that ribosomal frameshifting between Gag‐Pol may be responsible for rescue of the gRNA from mRNA degradation pathways and their subsequent packaging. Challenging this notion, the Hu group (Nikolaitchik & Hu, 2014) analysed this aspect in the context of deletion or mutations in ribosomal frameshifting signals (RFS) in proviruses [as opposed to a less than ideal dual complementation system used by Chamanian *et al*. (2013)]. Data from the Hu group suggests that while ribosomal frameshift signals may be important for the successful virus lifecycle, they are not *per se* directly involved in the process of RNA packaging since all RFS mutants examined were efficient in RNA packaging and generated viral titres similar to those observed with the wild type virus (Nikolaitchik & Hu, 2014).

However, many other observations support the significance of Gag/Pol precursors to successful infectious virion formation. For instance, in MLV, when Pol is supplied in *trans*, the resulting RT activity (a measure of virion production since the RT enzyme is packaged into the virus particle) is lower compared to that produced from wild‐type Gag‐Pol expression where Pol is expressed in *cis*. Additionally, particles generated under these conditions are slightly less infectious than those formed by the wild type virus, highlighting the importance of Gag‐Pol fusion protein in assembling fully functional and infectious MLV particles (Buchschacher *et al*., 1999). Similarly, introducing mutations that enhance or abrogate ribosomal frameshifting in HIV‐1 have been shown to result in a ~50% reduction in RNA packaging when expression was restricted to Gag alone (Benner *et al*., 2022). However, when Gag‐Pol was provided in *trans*, RNA packaging was restored to wild‐type levels, reinforcing the role of Gag‐Pol in this process (Benner *et al*., 2022). Furthermore, the IN domain of HIV‐1 Pol has been shown to interact with gRNA, a critical aspect required for the proper morphogenesis of mature viral particles (Elliott *et al*., 2020; Engelman, 1999; Jing *et al*., 2025; Jurado *et al*., 2013; Kessl *et al*., 2016; Liu *et al*., 2021). Moreover, maintaining the wild type ratio of Gag to Gag‐Pol precursors has been shown to be important for not only HIV‐1 RNA dimerization, but also for viral infectivity, with Pol products such as RT and IN influencing the conformation of the gRNA dimer (Shehu‐Xhilaga, Crowe & Mak, 2001; Shehu‐Xhilaga *et al*., 2002). Similarly, mutations in the dimerization initiation site (DIS) of HIV‐1 gRNA have been shown to disrupt Gag maturation (Liang *et al*., 1998). Altogether, these findings suggest that gRNA not only regulates protease activity but also interacts with Gag‐Pol. Based on these observations, it is reasonable to propose that Gag‐Pol (in HIV‐1 and FIV) or Gag‐Pro and Gag‐Pro‐Pol (in MMTV and MPMV) contribute to the selection and packaging of gRNA. Therefore, comprehensive and systematic studies are needed to thoroughly investigate the roles of Gag‐Pol, Gag‐Pro, and Gag‐Pro‐Pol precursors across diverse retroviruses in selectively recognizing and packaging gRNA while discriminating against viral spliced and cellular RNAs. Such studies should allow the identification of general rules underlying retroviral genome expression and packaging, as well as mechanisms that are specific to a particular retrovirus.

### (5) Role of unpaired or single stranded purines (ssPurines) in the recognition of packaging signal sequences

Purines are one of the two major classes of nitrogenous bases in RNA and DNA and include adenine (A) and guanine (G), which are distinguished by their larger, two‐ringed aromatic structures. The larger aromatic surfaces of purines promote stronger base‐stacking interactions than pyrimidines, favouring protein recognition at exposed RNA sites (Allain *et al*., 1996; Bloomfield, Crothers & Tinoco, 2000). In structured RNAs, purines are frequently enriched in a single‐stranded fashion where they are solvent‐exposed and conformationally flexible, making them favourable substrates for protein recognition (Leulliot & Varani, 2001). Retroviral Gag, and specifically its NC domain, preferentially binds such unpaired or single‐stranded purines (ssPurines) because their extended aromatic surfaces support base‐stacking and hydrogen‐bonding interactions with conserved aromatic and basic residues within the NC zinc knuckles (De Guzman *et al*., 1998; Amarasinghe *et al*., 2000). Structural and biochemical analyses of HIV‐1 Ψ RNA demonstrate that NC recognizes single‐stranded, purine‐rich bulges rather than base‐paired helices, and mutational disruption of these exposed purines reduces RNA packaging efficiency (D'Souza and Summers, 2005; Keane *et al*., 2015). Genome‐wide crosslinking studies further show that Gag preferentially contacts A‐ and G‐rich motifs *in vivo*, reinforcing the idea that purine exposure contributes to selective genomic RNA recognition during assembly (Kutluay *et al*., 2014). This preference for exposed purines is consistent with broader principles of RNA–protein interactions, in which unpaired A/G rich motifs frequently act as recognition elements in ribonucleoprotein assembly, RNA trafficking, and viral genome selection (Kutluay *et al*., 2014; Jankowsky & Harris, 2015).

Unpaired or ssPurines also play a crucial role in the recognition of packaging sequences among retroviruses, aiding in the selective incorporation of viral RNA into assembling virions. These unpaired purines contribute to RNA structural flexibility, promoting specific interactions with viral Gag proteins and enhancing the affinity for key recognition motifs within the packaging signal. By stabilizing unique RNA conformations, ssPurines facilitate efficient genome selection, ensuring that only full‐length unspliced RNA is packaged while excluding host/non‐viral as well as viral spliced RNAs. Indeed, the presence of a stretch of purines in the packaging sequences on retroviral gRNA has been proposed to facilitate RNA packaging by functioning as a potential Gag binding site (Ali *et al*., 2016; Dubois *et al*., 2018; Lever, 2007; Lu *et al*., 2011b; Maldonado & Parent, 2016).

In the case of MMTV, nine phylogenetically conserved ssPurines (5′ GGAGAAGAG 3′) are present upstream of the mSD in the form of an apical loop in close vicinity of DIS within the bifurcated SL4 (Fig. 4; Aktar *et al*., 2014; Mustafa *et al*., 2018). The importance of this ssPurine stretch in MMTV gRNA packaging was established through mutational analyses. Deletion or substitution of ssPurines with pyrimidines resulted in the abrogation of gRNA packaging, suggesting that ssPurines play a crucial role and may function as a potential Gag binding site during MMTV gRNA packaging (Mustafa *et al*., 2018). This assumption was later confirmed through *in vitro* biochemical studies, such as footprinting, which revealed that ssPurines exhibit strong and specific binding to the full‐length MMTV Gag (Pr77^Gag^). Interestingly, MMTV Gag also demonstrated strong and specific binding to nucleotides within the PBS located in SL2, at a considerable distance from the ssPurines. Furthermore, simultaneous Gag binding to nucleotides in both ssPurines and PBS was found to be essential for enhancing MMTV gRNA packaging, highlighting, for the first time, the role of PBS in retroviral gRNA packaging (Chameettachal *et al*., 2021). Additionally, some mutants that retained efficient Gag binding to ssPurines and PBS owing to the preserved structures of SL4 and SL2 failed to package MMTV RNA. This suggests that mere Gag binding to nucleotides within ssPurines and PBS is insufficient to mediate gRNA packaging; rather, it must occur while maintaining the native structure of the entire packaging sequence (further discussed later).

**Fig. 4:**
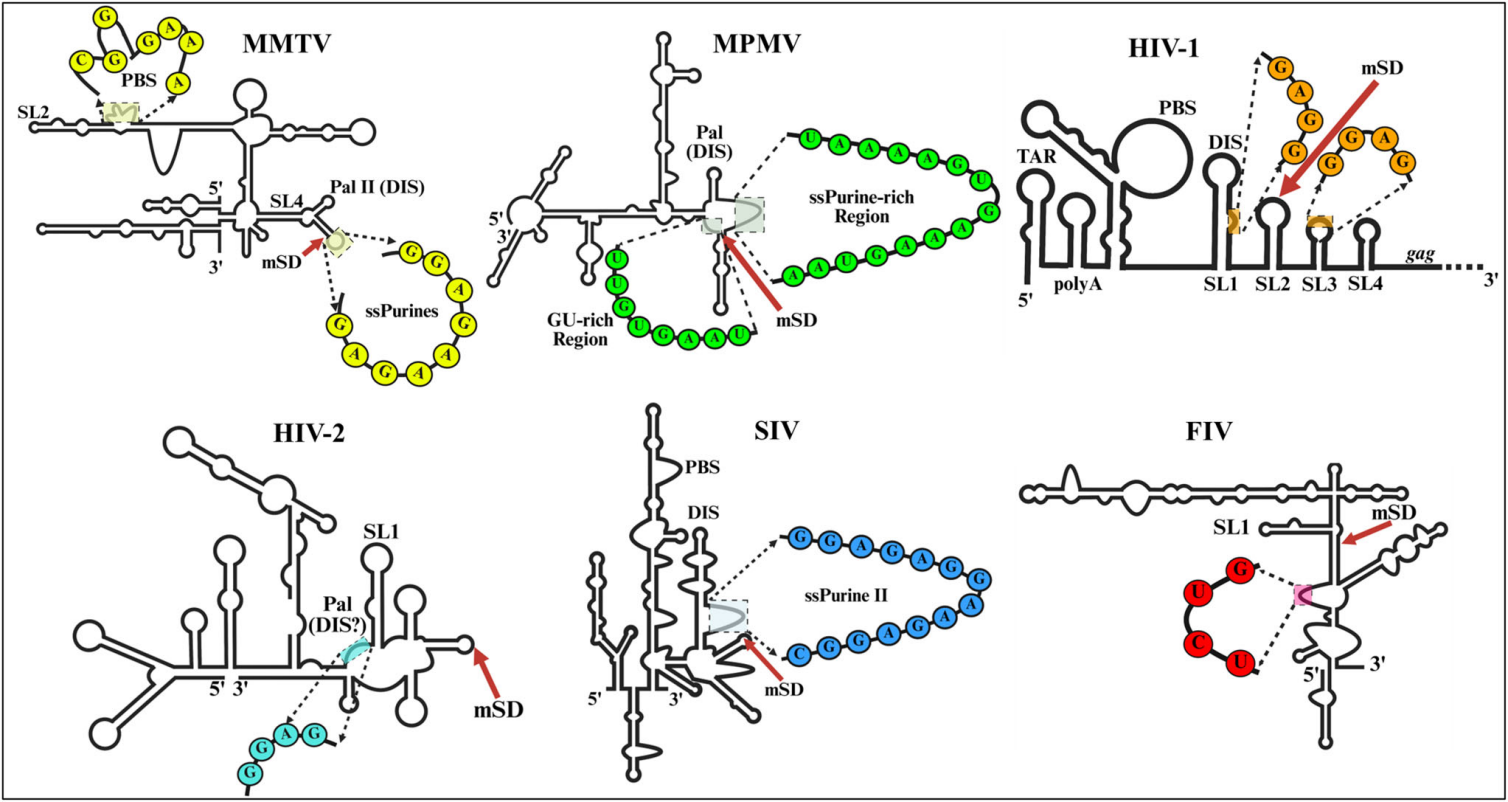
Schematic representation of the secondary RNA structures of the packaging signal RNA (*Psi*; ψ) in different retroviruses. The location of the unpaired or single‐stranded purines (ssPurines) within *psi* are enlarged and shown in colour. These nucleotides have been shown to be the primary Gag binding site(s), facilitating selective incorporation of unspliced RNA into assembling virions. In the case of feline immunodeficiency virus (FIV), unlike purines, the unpaired stretch of UCUG has been reported to be the potential Gag binding site. DIS, dimer initiation site; HIV‐1 and HIV‐2, human immunodeficiency virus types 1 and 2; MMTV, mouse mammary tumour virus; MPMV, Mason‐Pfizer monkey virus; mSD, major splice donor; Pal, palindromic motif; PBS, primer binding site; SIV, simian immunodeficiency virus; SL1–4, stem loops 1–4.

Since both ssPurines and PBS are architecturally positioned upstream of the mSD, they are present in both unspliced and spliced RNAs, raising the question of how only unspliced RNA gets selectively packaged over the spliced RNA despite harbouring ssPurines and PBS. This dilemma was later resolved through further *in vitro* biochemical studies, which confirmed that ssPurines in unspliced MMTV RNA remain unpaired and exhibit strong, specific binding to full‐length MMTV Gag (Chameettachal *et al*., 2021). In contrast, in spliced RNAs (both *env* and *sag*), ssPurines do not maintain an unpaired conformation (they are base paired with neighboring sequences) rendering them incapable of binding to Gag (Chameettachal *et al*., 2021). Such a scenario offers a plausible mechanism by which Gag preferentially binds unspliced RNA over spliced RNA, ensuring the strict selection of unspliced gRNA during MMTV packaging.

MPMV gRNA packaging has been shown to depend on a relatively large ssPurine‐rich region (5′ UUAAAAGUGAAAGUAA 3′) located upstream of the mSD (see the green nucleotides in Fig. 4). Interestingly, this region is the largest ssPurine‐rich stretch identified among all major retroviruses studied thus far, containing 75% purines and, like MMTV, positioned near the DIS. Eight nucleotides within this region (5′ GAAAGUAA 3′) are repeated as base‐paired purines located opposite to the ssPurines on the unspliced RNA, downstream of the mSD (Aktar *et al*., 2013; Jaballah *et al*., 2010). Initial genetic and biological analyses showed no substantial effect on MPMV gRNA packaging when ssPurines or the base‐paired purines were deleted individually. However, the combined deletion of both purine stretches nearly abolished gRNA packaging completely, suggesting a redundant role for these sequences and their potential function as Gag binding sites during MPMV gRNA packaging (Ali *et al*., 2020). Subsequent biochemical and structural studies revealed that full‐length MPMV Gag selectively and preferentially binds to nucleotides within two opposing loops: one binding site comprising within the ssPurine‐rich region and the other GU‐rich region (5′ AAGUGUU 3′; out of which AA are the last two nucleotides of the repeat region) downstream of the mSD. These findings support the initial hypothesis of functional redundancy between the two purine regions in MPMV gRNA packaging (Ali *et al*., 2020; Pitchai *et al*., 2021).

These observations created a challenge similar to that observed in MMTV: the ssPurine‐rich region that binds MPMV Gag is also present in spliced RNA, raising the question of how the selectivity towards packaging only the unspliced RNA is achieved in MPMV. This discrepancy was resolved through structural studies, which demonstrated that in MPMV *env* spliced RNA, a portion of the ssPurine region, the tip of the loop (5′ AGUGA 3′) adopts a base‐paired conformation (Pitchai *et al*., 2021). Notably, four of these five nucleotides in their base‐paired form have been shown to exhibit strong Gag‐binding affinity (Fig. 4). This structural arrangement likely restricts Gag binding to unspliced RNA, thereby ensuring the specific and preferential selection of unspliced RNA for packaging. This begs the question of how these two Gag binding sites function in MPMV unspliced RNA. A plausible scenario that has been proposed is that Gag first bind to the ssPurine‐rich region, distinguishing MPMV RNA from cellular mRNAs (Pitchai *et al*., 2021). This initial binding is followed by interaction with the second binding site (5′ AAGUGUU 3′), promoting Gag multimerization onto the unspliced RNA. Since unspliced RNA contains both Gag binding sites, while spliced RNA either lacks them or retains them in a base‐paired conformation, this allows Gag to specifically recognize and package unspliced RNA into assembling virions while leaving behind cellular and spliced RNAs.

The role of purine‐rich regions in complex retroviruses such as HIV‐1, HIV‐2, and human T cell leukemia virus type 1 (HTLV‐1) appears to be more complex than initially thought (Abd El‐Wahab *et al*., 2014; Baig, Lanchy & Lodmell, 2009; Bernacchi *et al*., 2017; Comas‐Garcia *et al*., 2018; De Guzman *et al*., 1998; Ding *et al*., 2020; Dubois *et al*., 2018; Keane *et al*., 2015; Keane & Summers, 2016; Kenyon *et al*., 2015; Nikolaitchik *et al*., 2020; Singh *et al*., 2018; Umunnakwe *et al*., 2021; Wu *et al*., 2018). In HIV‐1, gRNA packaging is facilitated by a G//AGG internal loop within SL1, located close to the DIS. This loop serves as a primary Gag binding site (Fig. 4; Abd El‐Wahab *et al*., 2014), but like the ssPurine sequences of MMTV and MPMV, it is present in both unspliced and spliced RNAs, raising the same question of how unspliced RNA is preferentially selected over spliced RNAs for packaging in HIV‐1. To address this, a dual regulatory mechanism based on steric hindrance has been proposed, explaining the selective binding of Gag to the G//AGG internal loop in the gRNA (Abd El‐Wahab *et al*., 2014). According to this model, an upstream region of SL1 (TAR, polyA and PBS) present in all HIV‐1 RNAs impedes Gag binding to SL1, but this repressing effect is compensated by sequences downstream (nt 355 to 400) of SL4, exclusively present in the gRNA.

In the case of HIV‐2, a 5′ GGRG 3′ (R refers to purine, i.e. A or G) motif upstream of the DIS has been identified as an essential element for RNA packaging and is thought to function as a Gag binding site (Baig *et al*., 2009). Recent findings further support this, confirming that the GGAG motif plays a key role in HIV‐2 RNA packaging (Fig. 4; Umunnakwe *et al*., 2021). In HTLV‐1, NC binding preferences also exhibit a strong affinity for guanine residues (Wu *et al*., 2018). However, structural probing employing selective 2′‐hydroxyl acylation analysed by primer extension (SHAPE) revealed that out of the nine guanosine residues available, only four were protected by NC binding (Wu *et al*., 2018). These findings suggest that while purine‐rich regions play a significant role in RNA packaging across complex retroviruses, the mechanisms governing their specificity and function vary between different viruses.

Furthermore, studies in HIV‐1 and HIV‐2 indicate that, in addition to primary Gag binding sites, which, when mutated, result in a substantial decline in RNA packaging, multiple secondary and tertiary Gag binding sites also contribute towards gRNA packaging. While individual mutations in these secondary or tertiary sites only minimally affect RNA packaging, their combined disruption alongside mutations in the primary Gag binding sites results in a significant, additive loss of packaging efficiency (Nikolaitchik *et al*., 2020; Umunnakwe *et al*., 2021). Thus, mutations in either HIV‐1 SL1 or SL3 alone lead to only a mild to moderate reduction in gRNA packaging. However, concomitant mutations in both SL1 and SL3 result in severe defects in RNA packaging (McBride, Schwartz & Panganiban, 1997). Similarly, a biologically relevant study employing competitive packaging conditions demonstrated that rather than relying on a single, specific Gag binding site, multiple guanosine residues act cooperatively to enhance Gag binding and facilitate RNA packaging (Nikolaitchik *et al*., 2020). Footprinting experiments have provided further insights, revealing that HIV‐1 Gag binds to secondary and tertiary binding sites with varying affinities, ranging from moderate to low (Abd El‐Wahab *et al*., 2014; Keane & Summers, 2016). In the case of MLV, given the fact that the Gag polyprotein binds more selectively to RNA packaging sequences compared to NC alone, this observation suggests that the specificity and strength of nucleotide interactions may depend on the full‐length Gag rather than exclusively on its cleaved NC domain (Gherghe *et al*., 2010). These observations emphasize the idea that HIV‐1 and HIV‐2 employ a multifaceted RNA packaging strategy that integrates multiple binding elements with varying efficiencies to ensure efficient genome selection.

In SIV, four distinct ssPurines loops have been reported. Two of these, ssPurine I (5′ AAAGG 3′) and ssPurine II (5′ GGAGAGGAAGAGGC 3′, located near the DIS), are positioned upstream of the mSD, while the remaining two, ssPurine III (5′ ACAAAAAAGAAAUAG 3′) and ssPurine IV (5′ AGGAAG 3′), are positioned downstream of the mSD. Functional analysis through mutational studies has shown that among these, only ssPurine II is critical for efficient SIV gRNA packaging, whereas ssPurines I, III, and IV have little to no impact on SIV gRNA packaging (Pillai *et al*., 2021). Disruptions in ssPurine II loop, whether through deletion or substitution, lead to its base pairing, which in turn abolishes RNA packaging. This finding underscores the importance of both the sequence and the looped conformation of ssPurine II in facilitating proper packaging of the viral genome (Fig. 4; Pillai *et al*., 2021). The experimental data further underscores that, unlike MMTV, MPMV, and HIV‐1, which possess multiple nucleocapsid/Gag binding sites, SIV depends on a single stretch of unpaired purines for its RNA packaging. Therefore, the scenario in the case of SIV closely resembles that of HIV‐2, where a single unpaired purine sequence (5′ GGAG 3′) serves as the main Gag binding site. Altering the guanines within this purine motif significantly impact RNA packaging, highlighting the crucial role of unpaired guanosines in the RNA packaging process of HIV‐2 (Fig. 4; Umunnakwe *et al*., 2021). Although biological data and structural prediction analyses highlight the significance of ssPurines II in SIV gRNA packaging, additional RNA–protein interaction studies are needed to clarify whether ssPurine II serves as a primary Gag binding site or not. Furthermore, if it does play this role, an important question remains: how does SIV ensure that spliced RNAs, which also contain ssPurine II, are excluded from packaging? Addressing this will be crucial for fully understanding the molecular mechanisms governing selective genome packaging in SIV.

In FIV, unlike other retroviruses discussed above, a four‐nucleotide unpaired stretch of UCUG plays a crucial role in gRNA packaging. Although not purine‐rich, all four nucleotides work synergistically to facilitate FIV gRNA packaging and serve as a putative binding site for full‐length Gag (Pr50^Gag^) (Fig. 4; Krishnan *et al*., 2023). This UCUG sequence is located upstream of the mSD, making it a part of both unspliced and spliced RNAs. Biochemical and structural analyses revealed that the spliced Env mRNA bound to FIV Gag with lower affinity, which was attributed to the fact that UCUG was base paired in the spliced Env mRNA. This base pairing likely reduces UCUG's accessibility to Gag, thereby favouring unspliced RNA for Gag binding during genome selection and packaging (Krishnan *et al*., 2023).

Interestingly, the UCUG motif is not unique to FIV and has been described as a Gag binding site in MLV as well. NMR studies have demonstrated that in MLV, the UCUG motif binds with high affinity to both the NC and full‐length Gag (D'Souza & Summers, 2004; Gherghe *et al*., 2010; Miyazaki *et al*., 2010). However, in MLV, the UCUG motif is base‐paired in the monomeric RNA conformation, necessitating RNA dimerization. It is only in the dimeric RNA conformation that the UCUG becomes unpaired and readily accessible for Gag binding. Further footprinting experiments using longer RNA sequences have identified two such motifs (UCUG‐UR‐UCUG), both of which are essential for high‐affinity NC binding (Gherghe *et al*., 2010). As expected, the guanosine (G) residues in the UCUG‐UR‐UCUG motif interact with NC and Gag *via* the zinc knuckle domain (D'Souza & Summers, 2004; Gherghe *et al*., 2010; Miyazaki *et al*., 2010), confirming its role as an RNA‐binding motif.

### (6) Role of RNA–RNA long range interactions (LRIs) in retroviral gRNA packaging

RNA viruses rely on conserved structural information found in both noncoding and occasionally coding sequences to execute crucial events during their life cycle. The 5′ end of the genome in numerous RNA viruses, including retroviruses, harbours a wealth of *cis*‐acting information conveyed through diverse higher‐order structures. Long‐range RNA–RNA interactions have been observed between distinctive 5′ end nucleotides and those a few hundred to thousands of nucleotides downstream in positive‐sense RNA viruses. Recent studies have highlighted the significance of these long‐range RNA sequence interactions in playing a pivotal role in viral replication. These include functions such as translational recoding phenomena of ribosomal frameshifting and stop codon readthrough, which heavily depend on long‐range RNA–RNA interactions (Fricke & Marz, 2016; reviewed in Nicholson & White, 2014).

RNA structure predictions, combined with biochemical analyses such as SHAPE and phylogenetic survey of sequences involved in retroviral gRNA packaging have revealed the presence of LRIs harbouring complementary sequences at the 5′ ends of the viral genomes. In most cases, these LRIs are phylogenetically conserved among different strains of a particular retrovirus, although their length may vary between strains. Since the overall RNA secondary structure of retroviral packaging sequences is anchored by these LRIs, they may provide structural or sequence‐level stability, as observed in many retroviruses, including HIV‐1, FIV, MMTV, and MPMV (Figs 5 and 6; Abbink *et al*., 2005; Abbink & Berkhout, 2003; Aktar *et al*., 2014; Chameettachal *et al*., 2021; Kalloush *et al*., 2016; Keane *et al*., 2015; Kenyon *et al*., 2008; Krishnan *et al*., 2023; Paillart *et al*., 2002, 2004b; Prabhu *et al*., 2024; Rizvi *et al*., 2010; Siegfried *et al*., 2014; Smyth *et al*., 2015; Tran *et al*., 2015). Despite significant sequence and structural heterogeneities among human, simian, and feline *lentiviruses*, the persistence of LRIs in isolates of HIV‐1, HIV‐2, MPMV, MMTV, and FIV provides additional evidence for their functional importance in retroviral life cycle (Abbink & Berkhout, 2003; Kalloush *et al*., 2016; Kenyon *et al*., 2008, 2011; Krishnan *et al*., 2023; Paillart *et al*., 2002; Prabhu *et al*., 2024; Rizvi *et al*., 2010). The crucial role of LRIs is additionally underscored by the observations that mutations destabilizing the complementarity of these LRIs adversely affect crucial steps in the retroviral life cycle, including RNA packaging and dimerization (Abbink & Berkhout, 2003; Kalloush *et al*., 2016; Krishnan *et al*., 2023; Paillart *et al*., 2002; Prabhu *et al*., 2024; Rizvi *et al*., 2010).

**Fig. 5:**
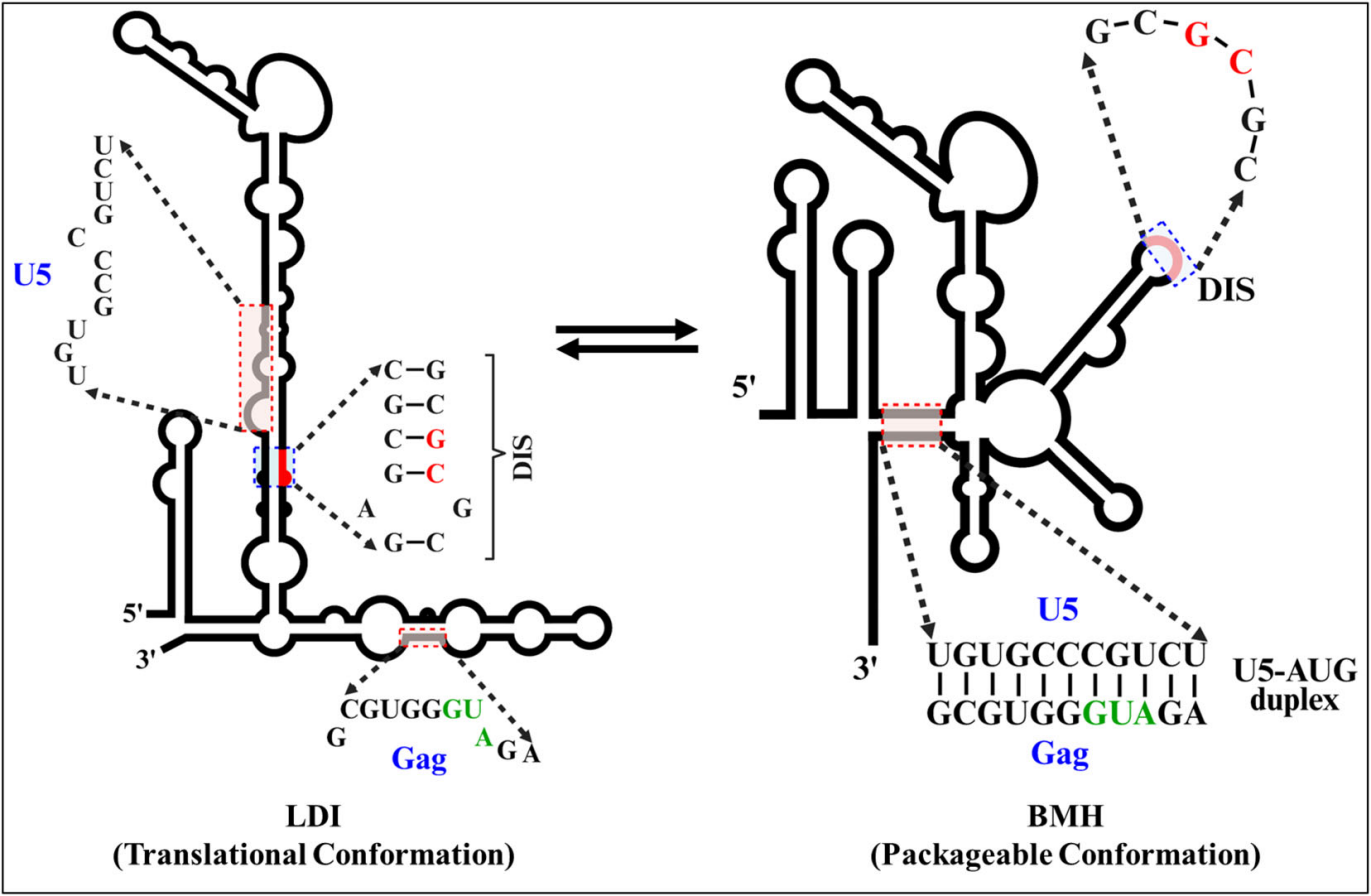
Schematic representation of the human immunodeficiency virus type 1 (HIV‐1) 5′ unique sequences (U5) that fold into two structural models: the long‐distance interaction (LDI) and branched multiple hairpin (BMH) conformations. The LDI conformation involves long‐range base‐pairing interactions masking the dimer initiation site (DIS) and packaging determinants while exposing the Gag start codon, promoting efficient translation while preventing dimerization and packaging. In the BMH ‘packageable’ conformation, several stem‐loop motifs are exposed, including the DIS, facilitating RNA dimerization which is a prerequisite for genome packaging. At the same time, the Gag start codon is sequestered within the 3D structure, thereby downregulating translation.

**Fig. 6:**
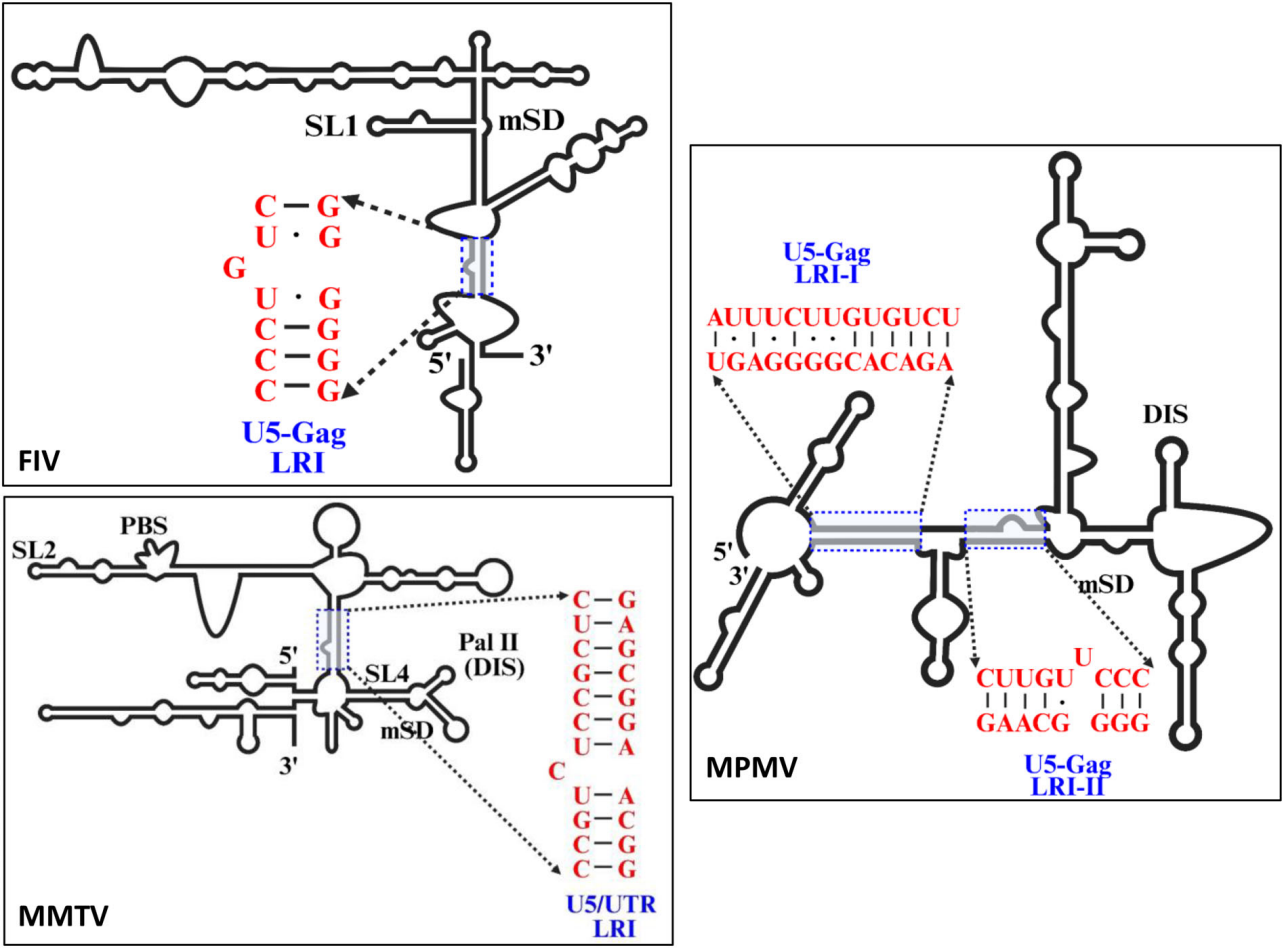
Schematic representations of the retroviral packaging sequences (*Psi*; ψ) featuring long range RNA–RNA interactions (LRIs) observed in different retroviruses highlighted as coloured and enlarged nucleotides. Since the overall RNA secondary structure of retroviral packaging sequences is anchored by these LRIs, they may provide structural or sequence‐specific stability to packaging sequences, underscoring their key role in gRNA packaging during viral replication. DIS, dimer initiation site; mSD, major splice donor; Pal, palindromic motif; SL, stem loop; U5, sequence unique to the 5′ end of the RNA genome; UTR, untranslated region.

In the case of HIV‐1, sequences within the 5′ UTR (~290 nucleotides) of the HIV‐1 RNA are highly conserved and contain essential regulatory elements that control various stages of the viral life cycle. It has been shown that this region can adopt two distinct secondary structures. One of these two conformations, called the Long‐Distance Interaction (LDI), in which the DIS is occluded due to intramolecular base‐pairing into a stem, while the Gag initiation codon is exposed and available for translational initiation (Fig. 5; Abbink *et al*., 2005; Abbink & Berkhout, 2003; Huthoff & Berkhout, 2001). The other conformation is called the Branched Multiple Hairpin (BMH), where the RNA is in a ‘packageable’ conformation and the DIS needed for RNA dimerization is open and available as a single‐stranded loop for RNA–RNA interactions; however, the Gag start codon is in an obscure position, not easily accessible for the initiation of translation. These structural variations also extend to the downstream region, which folds differently in each conformation. In LDI conformation, it adopts an extended hairpin, whereas in the BMH ‘packageable’ conformation, this segment forms a novel long‐range interaction with upstream U5 sequences, known as the U5‐AUG duplex, (Fig. 5; Abbink *et al*., 2005; Abbink & Berkhout, 2003; Huthoff & Berkhout, 2001). The two structural conformations, LDI and BMH align with their dual functional roles: (*i*) facilitating packaging into virions, a process that relies on RNA dimerization, and (*ii*) serving as a template for the translation of Gag and the Gag‐Pol fusion proteins.

Eventually an NMR‐based model for the BMH ‘packageable’ conformation was presented, demonstrating that binding of NC to this conformation is similar to the binding of full‐length RNA (Keane *et al*., 2015). The BMH ‘packageable’ conformation is further supported by the presence of several unpaired G residues, which have a strong affinity for the NC protein in the mature, infectious particles (Wilkinson *et al*., 2008). Additionally, studies have shown that substituting these G residues with A significantly reduces RNA packaging efficiency in assembling virions (Keane *et al*., 2015). This not only validates the BMH conformation as a packageable structure but further highlights the critical role of unpaired G residues in HIV‐1 RNA packaging, as discussed earlier. The importance of the U5‐AUG duplex as a key player in RNA packaging by modifying the structural arrangement of leader RNA sequences has been shown by deleting upstream sequences involved in this duplex formation, which results in RNA packaging defects, emphasizing its functional importance (Vicenzi *et al*., 1994). A recent study has elegantly shown that short‐ and long‐range interactions in the HIV‐1 5′ UTR regulate both genome dimerization and packaging. Specifically, a new interaction between PBS and SL1 has been proposed as a negative regulator of dimerization, Gag binding, and genome packaging (Ye *et al*., 2022). Finally, while phylogenetic analyses support the U5:AUG interaction by showing its conservation and co‐variation across HIV‐1 strains and related *lentiviruses* like SIV (Abbink & Berkhout, 2003; Damgaard *et al*., 2004; Keane *et al*., 2015; Lu *et al*., 2011b; Mueller, Das & Berkhout, 2016; Russell *et al*., 2003; Sakuragi *et al*., 2012; Song, Kafaie & Laughrea, 2008; Tran *et al*., 2015; Whitney & Wainberg, 2006), the LDI conformation has not been observed *in vivo*. Moreover, mutations designed to disrupt the LDI structure did not impact the translation of unspliced HIV‐1 RNA (Abbink *et al*., 2005).

A similar phenomenon of adopting alternative structural models discussed above for HIV‐1 has also been proposed for FIV packaging sequences, based on high‐throughput SHAPE (hSHAPE). This model not only confirmed the previously reported R/U5 and Gag LRI, but also suggested that the 5′ and 3′ sequences involved in forming LRI can also adopt an alternative conformation that occludes the DIS. As a result, this alternative structure is thought to promote translation and splicing over genome packaging (Kenyon *et al*., 2008, 2011; Rizvi *et al*., 2010). However, a later study, utilizing both SHAPE and genetic approaches, identified a different LRI. In this new model, the 5′ strand within U5 (5′ CCCUGUC 3′) remains the same as in the earlier LRI model (Kenyon *et al*., 2011), but instead of pairing with the previously reported Gag sequence (5′ GACAGGG 3′), it now forms base pairing with another sequence (5′ GGGGGG 3′) in the Gag region (Fig. 6; Krishnan *et al*., 2023). Notably, substituting the U5 and Gag sequences with each other while maintaining complementarity did not disrupt RNA packaging, further validating the existence and biological significance of the new R/U5 and Gag LRI (Krishnan *et al*., 2023). Irrespective of the exact nature of the sequences involved in the R/U5 and Gag LRI, these studies support the notion that the same RNA sequence can adopt different structures with functional consequences, making the study of RNA structure critical for deciphering its significance in biological systems.

The MMTV gRNA packaging sequences have also been shown to form an LRI composed of complementary sequences within the U5 region validated by SHAPE (Fig. 6; Prabhu *et al*., 2024). This LRI consists of an 11‐nucleotide‐long continuous stretch of complementary sequences within U5 (~200 nucleotides apart), exhibiting stable Watson–Crick base pairing. Therefore, it has been proposed that the secondary RNA structure of MMTV packaging sequences is anchored by this LRI, providing stability to the overall RNA secondary structure, which is crucial for MMTV gRNA packaging. Consistent with this, any perturbation designed to disrupt complementarity in this LRI severely compromises both MMTV RNA packaging and structural integrity, thereby validating the existence and biological significance of this LRI in the MMTV life cycle (Fig. 6; Prabhu *et al*., 2024). Furthermore, footprinting experiments have revealed that the MMTV full length gRNA efficiently binds to nucleotides within ssPurines and the PBS, which have previously been identified as the primary Gag binding sites in MMTV gRNA (Figs 4 and 7; Chameettachal *et al*., 2021). Conversely, in LRI non‐packaging mutants, the overall structure of MMTV packaging sequences is lost. Interestingly, Gag still binds, however, not to the previously identified primary Gag binding sites, as illustrated in the model shown in Fig. 7 (Prabhu *et al*., 2024). This finding challenges the long‐standing dogma that specific incorporation of retroviral gRNA into virus particles depends solely on the binding of Gag protein to packaging sequences in the genomic RNA. Instead, it is now becoming evident that Gag must bind to specific nucleotides within the correct three‐dimensional (3D) structural context to facilitate selective genomic RNA incorporation into virus particles. Thus, the 3D structure of the complexes formed between Gag and its packaging signal regulates viral particle assembly around the genomic RNA. Such 3D interactions help prevent or limit non‐specific incorporation of other viral and cellular RNAs, which also bind to Gag with high affinity (Fig. 7).

**Fig. 7:**
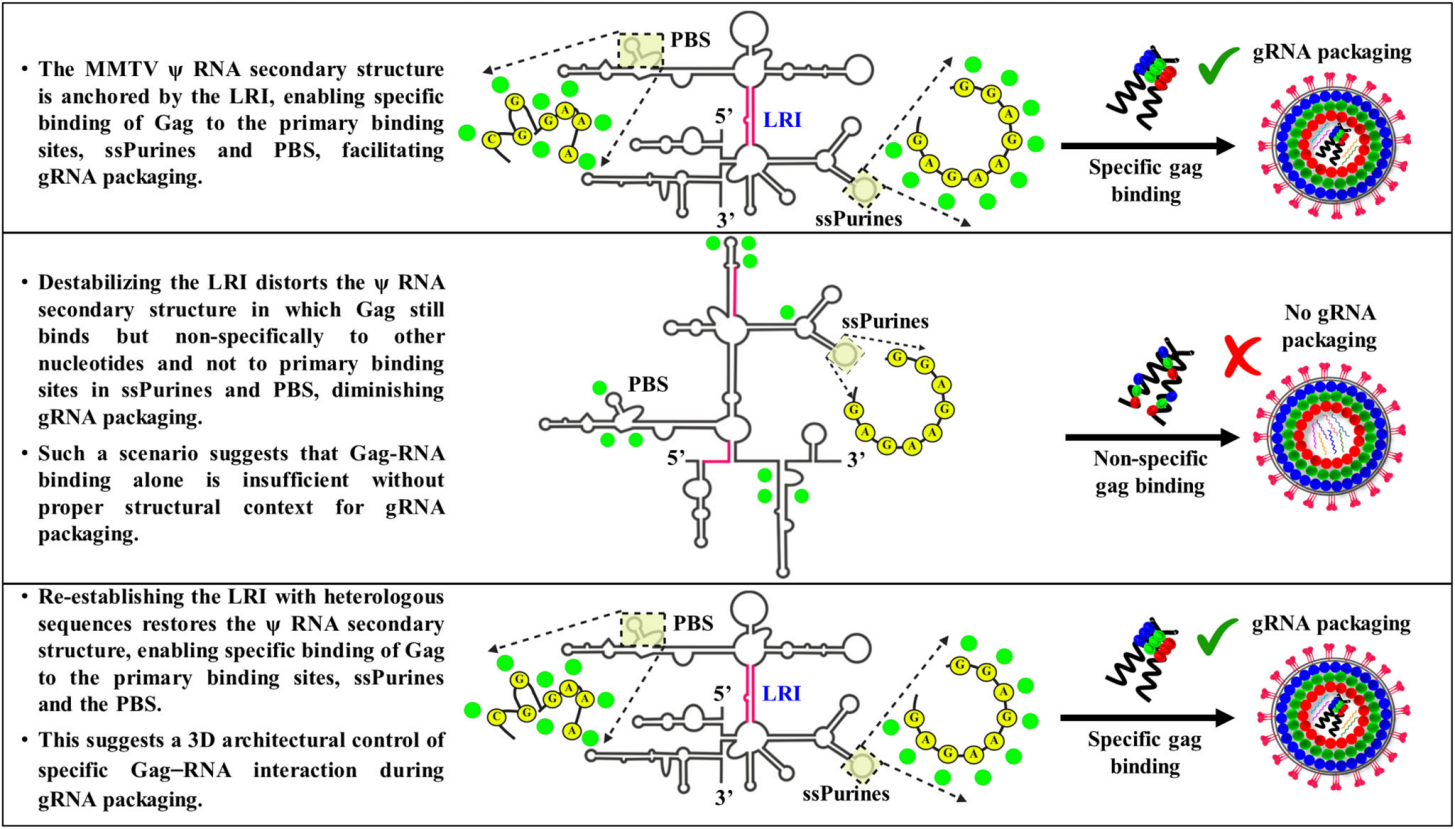
Model illustrating the role of long‐range RNA–RNA interaction (LRI) in modulating Gag (shown as green dots) binding to specific nucleotides within the packaging sequences to augment genomic RNA (gRNA) packaging. Based on a recent study (Prabhu *et al*., 2024), this model suggests that while Gag binding is essential for viral assembly, it is not sufficient for selective gRNA packaging. Effective incorporation of mouse mammary tumour virus (MMTV) gRNA requires Gag to recognize specific nucleotides within the correct three‐dimensional (3D) structural context. This structural specificity prevents non‐specific packaging of other viral and cellular RNAs that also bind Gag. PBS, primer binging site; ssPurines, single stranded purines; ψ, packaging signal.

Interestingly, the involvement of 11 nucleotides of U5 in forming the LRI in MMTV is analogous to HIV‐1, where 11 nucleotides of U5 in the BMH ‘packageable’ conformation form a long‐range interaction with Gag, known as the U5‐AUG duplex (Fig. 5; Abbink *et al*., 2005; Abbink & Berkhout, 2003; Huthoff & Berkhout, 2001). Similarly, MMTV resembles *lentiviruses* such as HIV and FIV, which possess a single identified long‐range RNA–RNA interaction (Prabhu *et al*., 2024; Abbink & Berkhout, 2003; Kenyon *et al*., 2008, 2011; Krishnan *et al*., 2023; Paillart *et al*., 2002; Rizvi *et al*., 2010). Finally, unlike other retroviruses, such as HIV‐1, HIV‐2, MPMV, and FIV, where U5/Gag sequences are involved in forming LRIs, MMTV LRIs involve only U5/UTR sequences (198 nucleotides apart) to achieve complementary base pairing (Prabhu *et al*., 2024; Abbink & Berkhout, 2003; Kalloush *et al*., 2016; Kenyon *et al*., 2008, 2011; Krishnan *et al*., 2023; Paillart *et al*., 2002; Rizvi *et al*., 2010).

In the case of MPMV, the gRNA packaging sequences contain two phylogenetically conserved complementary regions in U5 and Gag, known as LRI‐I and LRI‐II, which are in contrast to human, simian, and feline *lentiviruses* as well as MMTV harbouring only one identified LRI (Fig. 6; Abbink & Berkhout, 2003; Aktar *et al*., 2013; Kalloush *et al*., 2016; Kenyon *et al*., 2008, 2011; Krishnan *et al*., 2023; Paillart *et al*., 2002; Prabhu *et al*., 2024; Rizvi *et al*., 2010). A combination of structural probing techniques, such as SHAPE, and genetic analyses validated their existence and biological significance in the MPMV gRNA packaging process, highlighting their critical role in the retroviral life cycle (Kalloush *et al*., 2016). Notably, destabilizing LRI‐I completely abolishes MPMV RNA packaging, whereas restoring LRI‐I through heterologous sequence base‐pairing fully rescues RNA packaging. However, applying a similar genetic approach to LRI‐II failed to restore MPMV packaging, suggesting that while LRI‐I primarily serves a structural role, LRI‐II relies on specific sequence or contextual elements beyond simple base‐pairing (Fig. 6; Kalloush *et al*., 2016). These observations highlight the architectural as well as sequence‐specific role(s) of U5‐Gag LRIs in stabilizing the 5′ region of MPMV gRNA, reinforcing the broader importance of LRIs in retroviral RNA packaging beyond *lentiviruses*.

As can be seen, a wealth of studies conducted over the years unequivocally highlights the pivotal role of RNA–RNA and RNA–protein interactions in retroviral life cycle. These findings underscore the urgent need to deepen our understanding of long‐range intragenomic interactions, which hold immense potential for unveiling potentially promising therapeutic targets.

### (7) Retroviral RNA dimerization and its relationship to RNA packaging

Retroviruses uniquely package two copies of their gRNA in the form of a non‐covalent dimer. This conserved trait distinguishes retroviruses from many other RNA viruses and plays a central role in their replication cycle. Packaging of dimeric genome, although superficially redundant, is a deeply conserved evolutionary strategy that ensures selective encapsidation, genome integrity, promotes recombination (a key contributor to the genetic diversity and adaptability of retroviruses), and ensures that viral replication can proceed even if one RNA copy is damaged (Aagaard *et al*., 2004; Berkhout & van Wamel, 1996; Dubois *et al*., 2018; Houzet *et al*., 2007; Johnson & Telesnitsky, 2010; McBride *et al*., 1997; Mikkelsen *et al*., 2000; Moore *et al*., 2007, 2009; Mougel & Barklis, 1997; Paillart *et al*., 1996a). Packaging the genome in a dimeric form plays multiple roles in the retroviral life cycle because additionally it accelerates reverse transcription, allows repair of damaged RNA through template switching, and facilitates genetic recombination, especially in viruses like HIV‐1, ultimately enhancing adaptability and resistance (Chin *et al*., 2007; Onafuwa‐Nuga & Telesnitsky, 2009; Parent *et al*., 2000; Sakuragi, Sakuragi & Shioda, 2007).

The dimeric nature of retroviral RNA was first inferred in the 1960s through sedimentation analysis of virion RNA from RSV, MLV, and other type‐C retroviruses (Duesberg, 1968; Robinson, Robinson & Duesberg, 1967). These RNA molecules sedimented at a higher molecular weight than expected for monomeric species, suggesting a dimeric structure. Subsequent electron microscopy of the viral RNA revealed Y‐ or T‐shaped secondary structures consisting of two intertwined strands joined near their 5′ ends that were subsequently termed ‘rabbit ears’ or the dimer linkage structure (DLS; Bender *et al*., 1978; Bender & Davidson, 1976; Dube *et al*., 1976; Gonda, Rice & Gilden, 1980; Greatorex, 2004; Kung *et al*., 1976; Maisel *et al*., 1978; Murti, Bondurant & Tereba, 1981; Prats *et al*., 1990). DLS are structured motifs which are generally located at the 5′ end of the gRNA (Abbink & Berkhout, 2003; Aktar *et al*., 2013, 2014; D'Souza *et al*., 2004; Jossinet *et al*., 2001).

These studies later paved the way for identifying the DIS which truly delineated the underlying mechanism of the dimerization process, especially after the discovery of the HIV‐1 DIS. The DIS is a central player in retroviral RNA dimerization, typically located in the 5′ UTR, that initiates base pairing between two RNA molecules. In avian leukosis virus (ALV), MPMV, MMTV, and HIV‐1, a GC‐rich stretch of six nucleotides containing a conserved GC dyad has been identified as the DIS (Aktar *et al*., 2013, 2014; Clever, Wong & Parslow, 1996; Polge *et al*., 2000; see GC dyad highlighted in green in Fig. 8). Interestingly, in FIV, a 10‐nucleotide palindromic sequence located within the Gag coding region was proposed to serve this role (Kenyon *et al*., 2011). However, subsequent analyses suggested that this region may be involved in intermolecular base pairing during dimerization since these sequences were found to be base paired, casting doubt on its function in RNA dimerization (Krishnan *et al*., 2023).

**Fig. 8:**
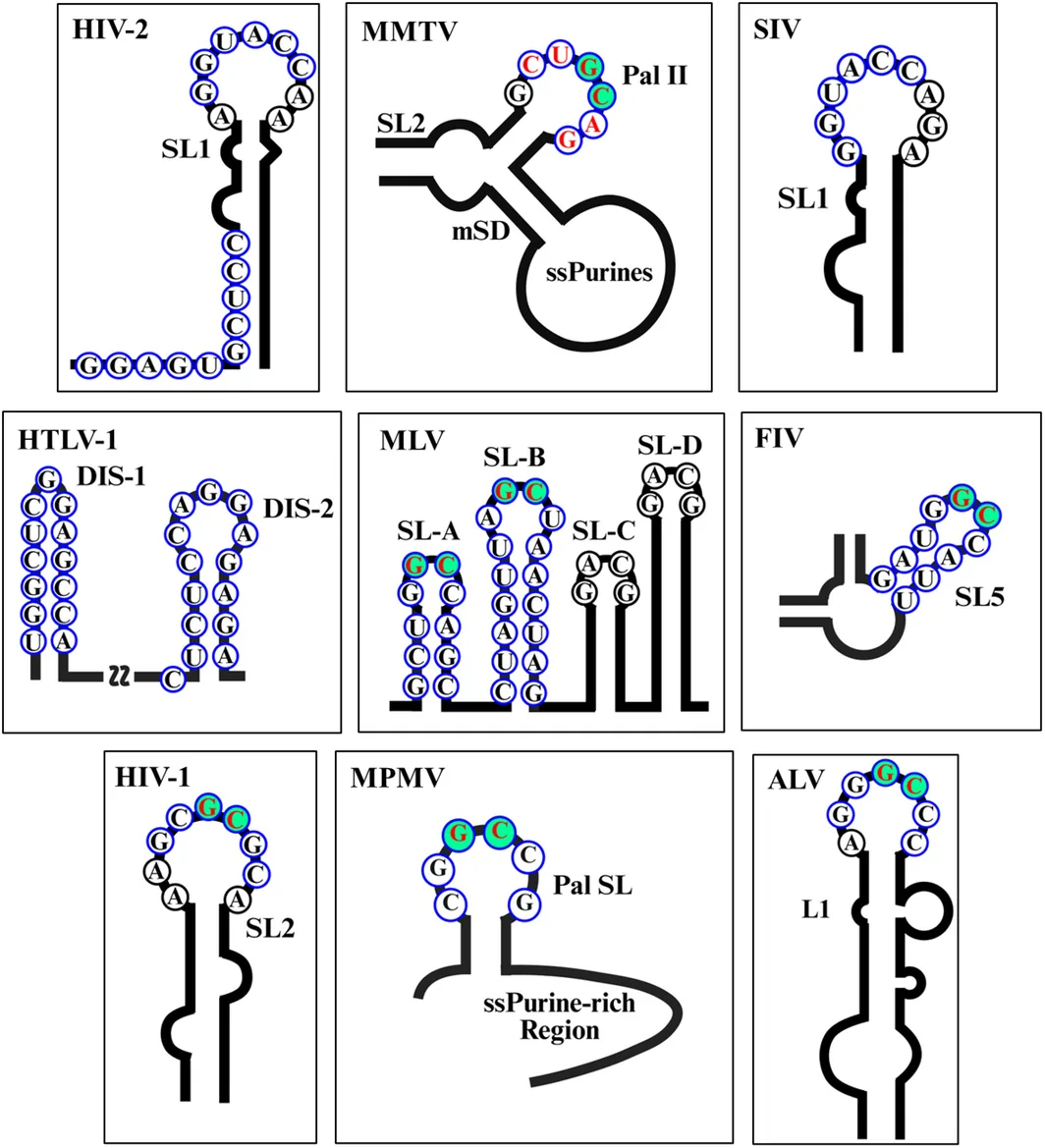
Schematic representation of the palindromic dimerization initiation site (DIS) across different retroviruses responsible for mediating the initial ‘kissing‐loop’ interaction between two copies of gRNAs. A conserved GC dyad, highlighted with a green background, is found in multiple strains of different retroviruses, such as mouse mammary tumour virus (MMTV), murine leukemia virus (MLV), feline immunodeficiency virus (FIV), human immunodeficiency virus type 1 (HIV‐1), Mason‐Pfizer monkey virus (MPMV), and avian leukosis virus (ALV). However, it is absent from other retroviruses, such as human immunodeficiency virus type 2 (HIV‐2), simian immunodeficiency virus (SIV), and human T cell leukemia virus type 1 (HTLV‐1). Nucleotides shown within blue circles correspond to either fully or partially palindromic sequences that are involved in the dimerization process. SD, major splice donor; Pal, palindromic motif; SL, stem loop; ssPurines, single stranded purines.

The DIS is especially prominent in HIV‐1, where a short palindromic sequence in a stem‐loop was found to initiate genome dimerization *via* base‐pairing with a complementary loop on a second RNA copy (Fig. 8; Laughrea & Jetté, 1994; Skripkin *et al*., 1994). DIS‐mediated dimerization was found to proceed through a ‘kissing‐loop’ interaction in which complementary loops of two stem‐loop structures base pair, thereby initiating RNA–RNA association (Fig. 8; Laughrea & Jetté, 1994; Paillart *et al*., 1994; Skripkin *et al*., 1994). In HIV‐1, the DIS is located within a short, thermodynamically stable stem‐loop structure in the 5′ leader region. This stem‐loop contains a 6‐nucleotide palindromic sequence at its apex (e.g. GUGCAC in subtype A; GCGCGC in subtype B; Fig. 8), flanked by conserved purine residues that play key roles in loop orientation and base stacking. The importance of DIS in the HIV‐1 life cycle has been underscored by mutagenesis studies, demonstrating that disruption of the palindrome abolishes dimerization and impairs genome packaging. However, introducing compensatory mutations can restore these processes (Berkhout & van Wamel, 1996; Clever *et al*., 1996; Laughrea *et al*., 1997; Paillart *et al*., 1997, 1996b; Skripkin *et al*., 1994). It is crucial to emphasize that although HIV‐1 typically employs a palindromic DIS for genome dimerization, the formation of the LDI conformer (discussed earlier and depicted in Fig. 5) driven by intramolecular base‐pairing between the 5′ UTR and the Gag AUG, serves as an important regulatory mechanism that controls the accessibility of the DIS and, consequently, the process of dimerization (Abbink *et al*., 2005; Abbink & Berkhout, 2003; Huthoff & Berkhout, 2001). Nearly two decades after these observations, newer studies suggest a different mechanism for HIV‐1 gRNA dimerization and packaging based on the heterogeneity of the transcriptional start site (Nikolaitchik *et al*., 2021; Yasin *et al*., 2024). Interestingly, this mechanism is inclusive of the earlier proposed BMH and LDI models, an aspect that is further elaborated upon in Section III.8, which pertains to the role of heterogeneity in retroviral RNA transcripts.

Dimerization of HIV‐2 gRNA resembles that of HIV‐1 in several ways but also exhibits unique regulatory features. The process involves multiple palindromic sequences, especially in the SL1 region of the packaging sequences and the TAR hairpin III, which initiate and stabilize dimer formation through loop–loop base pairing (Fig. 8; Dirac *et al*., 2001; Purzycka, Pachulska‐Wieczorek & Adamiak, 2011). Additional elements, like a palindromic sequence within the PBS have also been shown to contribute to HIV‐2 RNA dimerization (Jossinet *et al*., 2001; Lanchy *et al*., 2003b; Lanchy & Lodmell, 2002). Later it was shown that although the SL1 region in HIV‐2 resembles the HIV‐1 dimer DIS, it is not essential for HIV‐2 RNA dimerization. Instead, a self‐complementary sequence within the PBS was found to be crucial for dimer formation. Notably, Jossinet *et al*. (2001) proposed that HIV‐2 gRNA dimerization occurs *via* the classical kissing loop interaction in the cytoplasm prior to and during its encapsidation into the newly assembling particle. However, this interaction uses the *Nar*I site located within the PBS and not SL1 (as in HIV‐1), where the cellular tRNALys3 also binds, and this process does not require any viral proteins. This interaction leads to secondary intermolecular interactions along the length of the two dimerized gRNA substrates. Subsequently, the viral precursor Gag polyprotein and/or mature NC recognize the dimerized RNA to facilitate its encapsidation. More importantly, this leads to disruption of the ‘kissing loop’ interaction at the *Nar*I site, making it available for the tRNALys3 to bind to the PBS on the gRNA for further steps of the retroviral life cycle. In the meantime, the secondary interactions that were adopted earlier keep the two gRNA molecules dimerized. These findings offer new insights into how RNA dimerization and tRNA annealing may be coordinated within the cellular environment (Jossinet *et al*., 2001).

Recent studies have investigated the dimerization mechanisms of two *betaretroviruses*, namely MMTV and MPMV (Fig. 8; Aktar *et al*., 2013, 2014). Using SHAPE and dimerization assays, MMTV has been shown to dimerize through two palindromic motifs, one within the PBS (PBS‐Pal) and another, termed Pal II (5′ CUGCAG 3′; containing a canonical GC dyad), located in a bifurcated SL4 between the PBS and the Gag start codon. Pal II appears to play a dominant role in dimerization, as its disruption had a stronger effect than mutations in PBS‐Pal (Fig. 8; Aktar *et al*., 2014). In MPMV, the key DIS is a palindromic sequence that adopts a short hairpin structure called Pal SL. The central 6 nt (5′ CGGCCG 3′) of the palindromic sequence containing a canonical GC dyad play a crucial role in RNA dimerization. Furthermore, *in vitro* heterodimerization could be mediated by *trans*‐complementary sequences on two independent RNA molecules, further confirming that this sequence likely initiates gRNA dimerization by forming a kissing loop complex (Aktar *et al*., 2013). The identified dimerization elements are highly conserved across MMTV and MPMV strains, and their modification significantly impairs genome packaging and viral replication (Aktar *et al*., 2013, 2014).

*Gammaretroviruses* typically use multiple palindromic sequences to mediate RNA dimerization, with MLV being the best‐studied example. Its dimer linkage structure involves ~170 nucleotides and spans across four stem‐loops (SL‐A to SL‐D), each playing a role in dimer formation (Badorrek & Weeks, 2005; De Tapia *et al*., 1998; Girard *et al*., 1995; Prats *et al*., 1990; Roy *et al*., 1990). SL‐A and SL‐B contain key palindromes essential for initiating dimerization, while SL‐C and SL‐D harbouring GACG apical tetraloop, stabilize the structure through conserved loop–loop interactions (De Tapia *et al*., 1998; D'Souza *et al*., 2004; Gherghe & Weeks, 2006; Girard *et al*., 1995; Konings *et al*., 1992; Ly & Parslow, 2002). Over the years, it has become clear that dimerization results in structural rearrangements in the genome in a fashion that exposes previously hidden Gag binding sites to augment dimeric gRNA packaging. This has been well‐demonstrated in MLV, where RNA dimerization exposes a UCUG motif (base paired in monomeric form) critical for Gag binding (D'Souza & Summers, 2004).

In BLV, a *deltaretrovirus*, the DIS lies within the 5′ end of the genome, overlapping the U5 region, the PBS, and adjacent sequences, which also coincide with the packaging signal. Disruption of this region halts genome packaging and viral replication (Katoh, Yasunaga & Yoshinaka, 1993; Mansky, Krueger & Temin, 1995). In the case of HTLV‐1, another *deltaretrovirus*, a 32‐nucleotide sequence with a conserved 14‐nucleotide palindrome just upstream of the PBS serves as the DIS (Fig. 8; Greatorex *et al*., 1996; Monie, Greatorex & Lever, 2001; Monie *et al*., 2004). Deletion of HTLV‐1 DIS results in only a modest reduction in infectivity of blood mononuclear cells (Nikolaitchik *et al*., 2023), indicating that other factors likely contribute to the dimerization and packaging process (Le Blanc *et al*., 2000).

The detailed molecular mechanisms underlying RSV RNA dimerization, an *alpharetrovirus*, remain poorly understood, especially when compared to the more extensively characterized retroviruses, such as HIV‐1, MLV, MMTV, and MPMV. A recent study, however, identified a bipartite DIS within the 5′ UTR, comprising a GC‐rich 6‐nucleotide palindromic sequence and a second, non‐palindromic sequence (Liu *et al*., 2020). Interestingly, these two elements appear to be functionally redundant, and neither is essential for viral replication in cultured cells. This finding mirrors observations in HIV‐1, where the DIS was shown to be dispensable for replication in peripheral blood mononuclear cells (PBMCs) (Hill *et al*., 2003), suggesting the involvement of additional sequences or mechanisms in RNA dimerization.

Foamy viruses (FVs) differ significantly from other members of the *Retroviridae* family, most notably in their Gag polyprotein, which lacks the typical zinc finger domains and instead contain Gly‐Arg rich regions for nucleic acid binding (Linial, 1999; Yu *et al*., 1996). Reverse transcription in foamy viruses occurs late, leading to DNA‐containing viral particles (Linial, 1999; Yu *et al*., 1996). RNA dimerization in FVs involves three sites (SI, SII, SIII), with the SII region containing a conserved 10‐nucleotide palindromic sequence playing a central role (Cain *et al*., 2001; Park & Mergia, 2000). Disruption of this motif (SII) severely impairs dimer formation *in vitro* and leads to replication defects in cell culture, likely due to defects in reverse transcription, even though genome packaging remains unaffected (Cain *et al*., 2001; Park & Mergia, 2000). This contrasts with HIV‐1 and MMTV where manipulations in DIS sequence led to defects in gRNA packaging (Aktar *et al*., 2014; Berkhout & van Wamel, 1996; Laughrea *et al*., 1997, 1999; Paillart *et al*., 1996a).

As discussed extensively, retroviruses must carefully select and package two full‐length RNA genomes, a process intricately linked to RNA dimerization. Since improper dimer formation can disrupt key replication steps, its timing and cellular location are tightly controlled. The timing of retroviral RNA dimerization, whether it occurs before or after packaging, remains a topic of debate. Some studies have even suggested that individual HIV‐1 monomeric RNA genomes may be recruited first, with dimerization occurring later within the virion in a protease‐dependent manner (Shehu‐Xhilaga *et al*., 2001; Song *et al*., 2007). While findings from protease‐deficient virions and Gag mutants imply initial monomeric RNA packaging, this could reflect the instability of loose dimers during RNA extraction (Höglund *et al*., 1997). Moreover, the disruption of SL1 or the DIS often impairs genome packaging (Berkhout & van Wamel, 1996; Houzet *et al*., 2007; Moore *et al*., 2007; Paillart *et al*., 1996a), and structural comparisons between intracellular and virion RNA indicate minimal changes (Paillart *et al*., 2004b). Recombination studies (Moore *et al*., 2009) on the other hand, lend support to the prevailing view that dimerization is initiated in the cytoplasm, likely during genome recognition by the Gag precursor prior to virus release (Ferrer *et al*., 2016; Jouvenet, Simon & Bieniasz, 2009; Moore *et al*., 2007, 2009; Sardo *et al*., 2015). The dimer then undergoes a conformational maturation step that enhances its thermodynamic stability, a transition from a loose to a tight dimer (Lodmell *et al*., 2001; Marquet *et al*., 1991; Skripkin *et al*., 1994).

Retroviruses differ in the timing and location of genome dimerization, which in turn influences their genetic diversity. As mentioned above, in the case of HIV‐1, gRNA dimerization typically occurs in the cytoplasm and is later stabilized at the plasma membrane through interactions with Gag, promoting random pairing of genomes and increasing recombination potential (Chen *et al*., 2016). In contrast, MLV and RSV genomes dimerize in the nucleus, with MLV doing so co‐transcriptionally, often leading to the packaging of identical RNA copies and reduced genetic reassortment despite a comparable rate of template switching (Chen, Maldonado & Parent, 2021; Flynn *et al*., 2004; Kharytonchyk *et al*., 2005). Studies on MLV further support this nuclear dimerization model, showing that RNAs from nearby or identical loci preferentially form dimers (Maurel & Mougel, 2010; Rasmussen & Pedersen, 2006). Whether Gag plays a direct role in dimerization for MLV or RSV, as it does in HIV‐1, remains an open question.

Studies on retroviral RNA dimerization highlight a key concept that RNA structure is both informational and functional, not merely passive but actively governing the retroviral replication cycle. Unravelling the structural and functional dynamics of RNA dimerization deepens our understanding of retroviral replication and offers promising targets for the development of novel antiviral strategies.

### (8) Role of heterogeneity in retroviral RNA transcripts, cap sequestration, and epitranscriptomics in RNA packaging

Recent studies have demonstrated that HIV‐1 transcription initiates at multiple start sites, leading to the production of distinct unspliced RNA species, particularly 1G RNA and 3G RNA, differing by only a few nucleotides at their 5′ ends (Fig. 9). Despite their near‐identical sequences, these RNAs adopt distinct structural conformations that significantly influence their fate within the infected cell (Fig. 9; Brown *et al*., 2020; Ding *et al*., 2021; Kharytonchyk *et al*., 2016; Masuda *et al*., 2015; Nikolaitchik *et al*., 2021, 2023; Rawson *et al*., 2022; Ye *et al*., 2022). The 1G RNA is selectively packaged over 3G RNA primarily due to structural differences in the 5′ UTR which plays a critical role in RNA dimerization and Gag‐mediated genome packaging. These studies suggest that the 1G RNA adopts a conformation that exposes DIS and multiple guanosine‐rich Gag‐binding sites, thereby favouring its incorporation into assembling virions (Fig. 9). In contrast, the 3G RNA is more likely to fold into an alternative structure that sequesters these key elements, making it more prone to translation rather than packaging (Fig. 9; Brown *et al*., 2020; Kharytonchyk *et al*., 2016; Masuda *et al*., 2015; Nikolaitchik *et al*., 2021, 2023). Gag, the primary viral structural protein essential for RNA selection and packaging, preferentially binds to exposed unpaired guanosines in the 5′ UTR (Nikolaitchik *et al*., 2020; Umunnakwe *et al*., 2021), stabilizing the RNA and promoting its incorporation into virions. In the case of 1G RNA, the structural conformation facilitates stronger Gag interactions, leading to efficient packaging. Conversely, the structural constraints of 3G RNA limits its availability for packaging, likely directing it towards translation (Fig. 9; Brown *et al*., 2020; Kharytonchyk *et al*., 2016; Masuda *et al*., 2015; Nikolaitchik *et al*., 2021, 2023). The biological significance of the heterogeneity in transcriptional start site for HIV‐1 has been recently tested in tissue culture by monitoring the replication capability of mutant viruses that produce either 1G or 3G RNAs (Nikolaitchik *et al*., 2025). Second site revertant analysis of the viruses that arose upon replication revealed that these revertants had compensated for the introduced mutation and had better replication fitness than the original mutants. Furthermore, second site revertants had re‐acquired the ability to generate both the 1G and 3G transcripts. These results confirm that the ability of a retrovirus to generate transcripts with multiple start sites is biologically relevant and creates variants with better replication fitness due to their ability to generate transcripts with distinct functions central to virus replication.

**Fig. 9:**
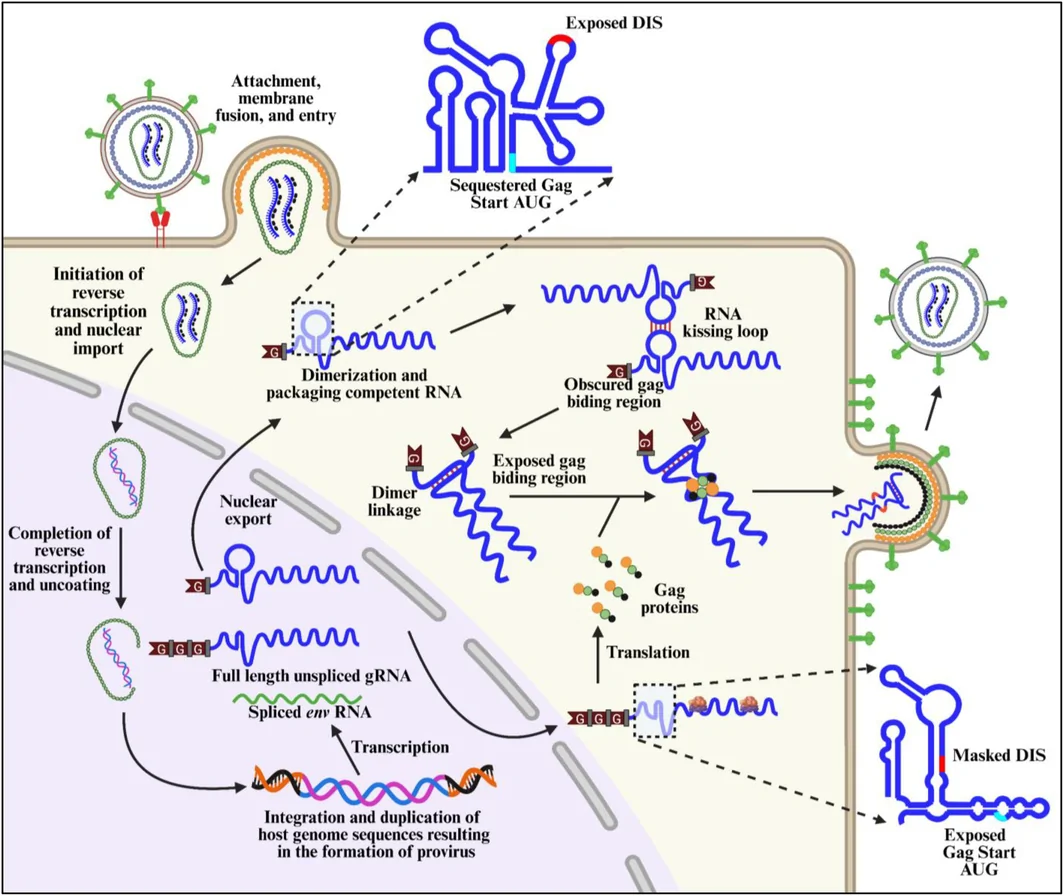
Schematic overview of the retroviral life cycle, highlighting the roles of distinct unspliced RNA species, particularly 1G and 3G RNAs, in human immunodeficiency virus type 1 (HIV‐1) genome dimerization and packaging. HIV‐1 transcription can initiate at multiple start sites, producing unspliced RNAs like 1G and 3G, which differ by only a few nucleotides at their 5′ ends. Despite such similarities, these RNAs adopt distinct structures that determine their fate: 1G RNA exposes the dimerization initiation site (DIS) and Gag‐binding sites, promoting its packaging into virions, while 3G RNA masks these elements and exposes the Gag initiation codon, making it more accessible for translation rather than packaging. gRNA, genomic RNA.

The preference for 1G RNA in packaging is not only observed in cell culture models but has also been validated in clinical samples from people living with HIV. Studies have shown that while 3G RNA is more abundant in infected cells, 1G RNA is significantly enriched in virions, reinforcing the notion that HIV‐1 employs a highly selective mechanism for gRNA packaging (Islam *et al*., 2025). The evolutionary advantage of this selective packaging mechanism likely lies in maintaining a balance between translation and genome packaging. By ensuring that only a specific subset of unspliced RNA is packaged, HIV‐1 optimizes its replication fitness and maintains a functional equilibrium between producing viral proteins and assembling infectious particles. Taken together, these studies demonstrate that 1G and 3G RNA have distinct functional roles, with the 5′ UTR structures playing a crucial part in determining their fate. Nevertheless, the precise mechanism that enables the preferential packaging of 1G RNA as the viral genome for packaging, while allowing 3G RNA to be more efficiently translated, remains obscure. Additional investigations are necessary to elucidate these processes, which could enhance our understanding of genome packaging, regulation of gene expression, and the complex control of HIV‐1 replication by diverse transcription start sites. Furthermore, SIV and HIV‐2 also exhibit the use of multiple transcription start sites and the selective packaging of a particular RNA species, suggesting that it may be a more common phenomenon used by retroviruses to control their replication (Duchon & Hu, 2024; Rawson *et al*., 2022). Since unspliced RNA serves as both the viral genome for packaging and a translation template across all retroviruses, understanding how different retroviruses regulate these dual functions is crucial. Thus, further studies are needed to uncover the role of transcription start site heterogeneity in this regulation across retroviruses to understand how retroviruses coordinate essential functions to ensure efficient replication.

In addition to structural factors, the host cell machinery plays a role in modulating gRNA packaging. For instance, cap sequestration has been implicated in this process, where the stability of the polyA stem and TAR elements may regulate the accessibility of the RNA cap for translation *versus* packaging (Ding *et al*., 2021; Ding & Summers, 2022). Sequestration of the cap structure may prevent translation initiation by cellular ribosomes, thereby making the RNA more available for packaging into virions. Additionally, it has been hypothesized that the host RNA processing and translation machinery can capture cap‐exposed RNA in an irreversible manner, further influencing its availability for packaging (Ding *et al*., 2021; Ding & Summers, 2022).

Employing biochemical, biophysical, and biological approaches, recent work puts all these disparate observations in the context of the flexibility of the RNA structure of the HIV‐1 leader region imposed due to heterogeneity of the transcription start site that leads to stabilization of the 5′ polyA sequence intricately involved in the decision to package or translate the RNA (Nikolaitchik *et al*., 2021; Yasin *et al*., 2024). These studies reveal that transcripts with 1G assume a branched multihairpin conformation and are preferentially packaged due to the presence of a stable 5′ polyA stem loop that is structurally stabilized by the TAR element. This leads to an exposed DIS, promoting RNA dimerization, resulting in its capture by the Gag polyprotein with a sequestered 5′ cap. On the other hand, the 3G‐containing RNA transcripts assume a structural conformation in which RNA elements crucial for both dimerization and Gag binding are masked, while those elements important for translation, such as the 5′ cap, are available, thus favouring translation over packaging. These studies certainly refine the molecular framework governing the choice between genomic RNA packaging and translation by demonstrating that transcription start site‐dependent exposure of the 5′ cap is a primary determinant. Nonetheless, the classical model proposed previously remains intact, that is RNA conformations that expose the DIS promote dimerization and packaging, whereas conformations that mask the DIS favour translation (Abbink *et al*., 2005; Abbink & Berkhout, 2003; Huthoff & Berkhout, 2001).

Epitranscriptomic modifications, particularly N^6^‐methyladenosine (m^6^A), also play a critical role in shaping HIV‐1 gRNA fate, influencing its processing, translation, and packaging. These chemical modifications introduce an additional regulatory layer that impacts viral gene expression, ultimately affecting replication efficiency and infectivity (Baek *et al*., 2024; Pereira‐Montecinos *et al*., 2022; Singh *et al*., 2022). Recent findings indicate that m^6^A modifications can alter translation dynamics, shifting the preference toward alternative cap‐binding mechanisms, such as the cap‐binding protein 80 (CBP80)/nuclear cap‐binding protein 3 (NCBP3) pathway, rather than the canonical eIF4‐dependent initiation, which may have implications for RNA packaging efficiency (Singh *et al*., 2022). Furthermore, the methylation state of unspliced HIV‐1 RNA appears to be a determinant of its function; m^6^A‐modified RNA favours Gag protein expression, while demethylated RNA is more efficiently packaged into virions (Pereira‐Montecinos *et al*., 2022). A more recent study, utilizing single‐molecule direct RNA sequencing, has revealed that HIV‐1 RNA contains specific m^6^A modifications predominantly near the 3′ end of the viral genome. These modifications influence RNA splicing, ensuring balanced production of unspliced, partially spliced, and completely spliced viral RNA species. The presence of m^6^A modifications suppresses excessive splicing, thereby maintaining adequate levels of unspliced RNA necessary for virion packaging (Baek *et al*., 2024). These findings suggest that HIV‐1 strategically exploits the epitranscriptomic landscape to fine‐tune its replication strategy, ensuring efficient gene expression while preserving genomic RNA integrity for packaging. Further research into the mechanisms governing m^6^A deposition and its interactions with viral and host proteins may provide new opportunities for antiviral therapeutic strategies targeting HIV‐1 at the RNA level.

### (9) Gag–RNA interactions serve as nucleation points for assembly and gag multimerization

As discussed extensively, the critical step of selective packaging of viral RNA genomes is mediated by specific interactions between the Gag polyprotein and high‐affinity binding sites within the packaging sequences of the gRNA (Abd El‐Wahab *et al*., 2014; Banerjee & Voth, 2024; Chameettachal *et al*., 2021; Clever *et al*., 1995; Krishnan *et al*., 2023; Lerner *et al*., 2022; Pitchai *et al*., 2021; Rein, 2019). However, *in vitro* studies have shown that under physiological conditions, Gag binds both cellular and viral RNA with nearly identical affinity (Comas‐Garcia *et al*., 2017; Rye‐McCurdy *et al*., 2014; Webb *et al*., 2013). Interestingly, increasing ionic strength in experimental settings significantly reduces nonspecific binding, allowing the identification of specific high‐affinity binding sites within the packaging sequences (Abd El‐Wahab *et al*., 2014; Chameettachal *et al*., 2021; Krishnan *et al*., 2023; Pitchai *et al*., 2021; Prabhu *et al*., 2024). Adding to this complexity, a cell‐based cross‐linking‐immunoprecipitation sequencing (CLIPseq) study revealed that while HIV‐1 Gag exhibits a slight preference for viral RNA, the majority of cytoplasmic Gag molecules remain associated with cellular mRNAs and that HIV‐1 can package cellular mRNAs (Kutluay *et al*., 2014; Rulli *et al*., 2007). This indicates that Gag's RNA‐binding properties alone cannot fully explain selective packaging of HIV‐1 RNA, leaving the precise mechanism obscure.

One compelling hypothesis suggests that HIV‐1 gRNA packaging occurs at the plasma membrane, requiring Gag to multimerize on RNA to form a stable complex capable of successful gRNA packaging. Supporting this idea, studies using a complementation system that separates Gag's roles in particle production and in gRNA packaging, have demonstrated that initial Gag–RNA interactions are highly specific due to the packaging sequences within the gRNA. Consistent with this, a study using single‐molecule localization microscopy has also shown that Gag first binds gRNA in the cytoplasm before multimerizing at the plasma membrane (Yang *et al*., 2018). This initial Gag–RNA interaction is crucial for assembly, as NC deletion disrupts clustering and delays particle production without altering morphology. Therefore, Gag–RNA interactions serve as nucleation points for assembly, facilitating Gag recruitment with packaging sequences as the key nucleation site. However, as assembly progresses, Gag's RNA binding becomes more nonspecific. Furthermore, mutations in Gag that disrupt either its RNA‐binding ability or multimerization prevent gRNA packaging, while still allowing particle formation (Duchon *et al*., 2021). This highlights the necessity of both specific RNA binding and Gag multimerization for efficient gRNA packaging. Although Gag can interact with various RNAs, its assembly is significantly enhanced in the presence of specific RNAs containing packaging sequences. These specific Gag–RNA interactions not only function as a nucleation point for viral assembly but also promote Gag recruitment to facilitate particle formation (Comas‐Garcia *et al*., 2018; Dilley *et al*., 2017; Duchon *et al*., 2021; Nikolaitchik *et al*., 2013; Yang *et al*., 2018). Thus, particle assembly and selective gRNA packaging are dependent on nucleation, which is a rate‐limiting step occurring more efficiently on viral RNA containing packaging sequences, compared to RNAs that lack packaging sequences (Rein, 2019).

### (10) The initial site of gRNA selection for packaging

As discussed earlier, the location of dimerization varies among retroviruses and this impacts initially where Gag binds to gRNA (Chen *et al*., 2021; Dorman & Lever, 2000; Kharytonchyk *et al*., 2005; Levin & Rosenak, 1976; Maurel & Mougel, 2010). It was traditionally believed that Gag recognizes and binds gRNA in the cytoplasm or at the plasma membrane. However, since retroviral RNA is transcribed in the nucleus and Gag polyproteins from various retroviruses (RSV, FIV, FV, HIV‐1, MPMV, MMTV, MLV) have been detected in the nucleus, it is plausible that Gag interacts with gRNA at an early nuclear stage (Baluyot *et al*., 2012; Beyer *et al*., 2013; Garbitt‐Hirst, Kenney & Parent, 2009; Gudleski *et al*., 2010; Kemler, Saenz & Poeschla, 2012; Kenney *et al*., 2008; Müllers *et al*., 2011; Nash *et al*., 1993; Paris *et al*., 2018; Scheifele *et al*., 2002; Schliephake & Rethwilm, 1994). Consistent with this possibility, recent evidence suggests that RSV Gag interacts with newly transcribed gRNA within transcriptional condensates, demonstrating a potential connection between co‐transcriptional dimerization and selective packaging of gRNA (Fig. 2; Lambert *et al*., 2024; Lambert, Maldonado & Parent, 2025; Maldonado *et al*., 2020, 2023; Maldonado & Parent, 2025; Chen *et al*., 2025). Whether the RSV Gag protein associates with gRNA before or after dimerization is still unclear. This Gag–RNA interaction also promotes Gag multimerization (Gudleski *et al*., 2010). RSV Gag multimerization triggers a structural shift in Gag, exposing its nuclear export signal (NES) to allow interaction with the CRM1 export receptor, facilitating the nuclear export of the Gag‐gRNA complex across the nuclear envelope, and directing it to the plasma membrane for packaging (Gudleski *et al*., 2010; Kenney *et al*., 2008; Scheifele *et al*., 2002; Scheifele, Ryan & Parent, 2005). Thus, the initial site of gRNA dimerization has significant implications for the MLV RNA packaging process and nuclear dimerization facilitates the immediate recognition and binding of the Gag polyprotein to the dimerized gRNA, promoting efficient packaging.

Conversely, HIV‐1 gRNA dimerization occurs predominantly in the cytoplasm, resulting in a random assortment of homodimeric and heterodimeric gRNA pairs (Moore *et al*., 2009). Such cytoplasmic dimerization allows for greater diversity in the packaged genomes, potentially enhancing viral adaptability (Onafuwa‐Nuga & Telesnitsky, 2009). HIV‐1 also relies on cytoplasmic interactions between Gag and gRNA, which may involve additional regulatory mechanisms to ensure selective packaging. In line with this, it was believed that Gag first associates with gRNA in the cytoplasm or at the plasma membrane, where viral assembly and budding take place (Barajas *et al*., 2018; Chen *et al*., 2016; Jouvenet *et al*., 2006; Kemler *et al*., 2012; Milev, Brown & Mouland, 2010). However, this perspective shifted with the recent observation demonstrating that HIV‐1 Gag associates with unspliced HIV‐1 RNA to form ribonucleoprotein complexes at sites of transcription in the nucleus (Milev *et al*., 2010; Tuffy *et al*., 2020; Ukah *et al*., 2018). In addition to these observations, it is also possible that this interaction occurs in both the nucleus and cytoplasm, as these processes are not necessarily mutually exclusive. Consistent with this, it has been shown that cytoplasmic viral protein complexes in cytoplasmic RNA granules are associated with host factors, such as DEAD‐box helicase 6 (DDX6) and ATP Binding Cassette Subfamily E Member 1 (ABCE1), known for promoting capsid assembly (Barajas *et al*., 2018). In the case of FIV, it has been suggested that Gag–RNA interaction initiates at the nuclear envelope (Kemler *et al*., 2012). Therefore, to resolve these observations, future research needs to focus on targeted deletions in packaging sequences and mutations in Gag that affect viral RNA binding. This approach should provide definitive insights into the *cis*‐ and *trans*‐acting elements required for Gag's interaction with unspliced viral RNA, both in the nucleus as well as cytoplasm, ultimately settling existing controversies.

### (11) Virus assembly intermediates and retroviral RNA packaging

Emerging evidence suggests that the process of gRNA packaging occurs through a series of assembly intermediates within host‐derived stress granules as well as RNA granules, which serve as precursors for viral particle formation. A recent study has highlighted the role of liquid‐liquid phase separation (LLPS) in the cytoplasmic interactions between HIV‐1 Gag and gRNA. Specifically, the zinc finger domains within the NC region of Gag have been shown to facilitate the formation of ribonucleoprotein (RNP) complexes through phase separation, a process believed to support viral assembly by suppressing translation and promoting the formation of assembly intermediates (Monette *et al*., 2020). Similar mechanisms may be at play in HIV‐2 and HTLV‐1, as their NC domains also exhibit Zn^2+^‐dependent phase separation properties (Monette *et al*., 2020). Notably, the regulation of RNP formation appears to be influenced by Zn^2+^ concentration, with high levels of NC shifting the process toward the formation of Zn^2+^‐dependent stress granules. Conversely, depletion of Zn^2+^ results in gRNA mislocalization, either sequestering it in stress granules or leading to nuclear retention (Monette *et al*., 2020). This dynamic interplay between RNP complexes and stress granules suggests a potential regulatory mechanism in which the NC domain of Gag and Zn^2+^ levels govern gRNA trafficking and its accessibility to sites of virus assembly.

It has also been demonstrated that the initial interaction between unspliced HIV‐1 RNA and Gag occurs in the RNA granule‐derived 80S assembly intermediate, rather than in the smaller ~10S soluble Gag complex, suggesting a structured, stepwise progression toward complete virion assembly (Barajas *et al*., 2018). Thus, in both HIV‐1 and FIV, host factors ABCE1 and DDX are involved in capsid assembly and facilitate the transition of Gag–RNA complexes to later assembly stages, and are also associated with Gag within the RNA granule‐derived 80S assembly intermediate. These host proteins remain bound to Gag until capsid assembly is complete, at which point they dissociate, allowing the immature capsid to undergo budding and release (Dooher *et al*., 2007; Reed *et al*., 2012, 2018; Tanaka *et al*., 2016; Zimmerman *et al*., 2002). Additionally, the double‐stranded RNA‐binding host protein, Staufen1, has been found to colocalize with HIV‐1 Gag in the viral RNP complex, indicating a potential role in gRNA packaging. This further supports the hypothesis that host cellular components play a conserved role in retroviral RNA packaging (Abrahamyan *et al*., 2010; Cochrane, McNally & Mouland, 2006; Mouland *et al*., 2000). However, recent findings challenge the idea that all Gag‐containing RNA granules represent true assembly intermediates, as a significant proportion of these complexes appear to consist of lower‐order Gag oligomers associated with ribosomes, rather than mature virion precursors (Deng *et al*., 2021). Given these inconsistent findings, further studies are necessary to delineate the precise role of assembly intermediates and host‐granule‐derived proteins in gRNA packaging. Advanced imaging techniques and biochemical analyses could help determine whether these intermediates are part of the on‐pathway assembly process or merely transient complexes that do not contribute directly to virion formation. Understanding these mechanisms is crucial for developing therapeutic interventions that target early‐stage viral assembly and RNA packaging.

## IV. CONCLUSION

(1) Despite decades of research, key aspects of retrovirus replication remain incompletely understood, continuing to drive new investigations. This review has highlighted how retroviruses, while sharing core genomic features, have unique features that impact their replication strategies. Special focus is given to how retroviruses have evolved diverse mechanisms to selectively dimerize and package their genomic RNA.
(2) RNA dimerization serves not only as a prerequisite for packaging but also as a strategic mechanism to promote genetic recombination and evolutionary adaptability.
(3) The interplay between RNA structures, *cis*‐acting elements, and Gag or retroviral protein functions reveal a finely regulated system optimized for viral survival and adaptability. Recent findings reveal that packaging is governed not merely by RNA sequence, but by higher‐order structural organization that distinguishes unspliced gRNA from other RNAs.
(4) Single‐stranded purines, LRIs, RNA cap sequestration, and transcriptional start site heterogeneity contribute to a multilayered regulatory network, ensuring selective genome packaging. Emerging evidence that epitranscriptomic modifications, such as m^6^A, influence RNA fate adds another layer of complexity and therapeutic potential.
(5) Fundamental questions persist: Is the gRNA recognized by Gag alone or by Gag in the context of Gag/Pol precursors? Does encapsidation always occur in the context of a dimerized RNA for specificity? What are the temporal and spatial constraints that dictate gRNA recognition and packaging? Are there additional, undiscovered RNA motifs or RNA–protein complexes that dictate packaging specificity? To what extent do host factors actively shape this selectivity?
(6) Future studies employing single‐molecule imaging, high‐throughput RNA structure probing, and integrative structural biology will be instrumental in answering these questions. A deeper mechanistic understanding of retroviral genomic RNA packaging will not only enrich our knowledge of virus replication, but also aid in the rational design of novel antiviral therapies that target critical RNA–protein interactions which are active in these processes.

## AUTHOR CONTRIBUTIONS

M.A.J.: conceptualization, writing, original draft preparation, review, and editing. S.G.P., L.M.A. and V.N.P.: writing, review, and editing. F.M.: conceptualization, supervision, writing, review and editing, and funding acquisition. T.A.R.: conceptualization, supervision, writing, review, editing, project administration and funding acquisition.
